# Supplementary material for: Mild proteasomal stress improves photosynthetic performance in Arabidopsis chloroplasts
Source: Nat Commun. 2020 Apr 3;11:1662. doi: 10.1038/s41467-020-15539-8 (PMC7125294; doi:10.1038/s41467-020-15539-8)
Supplement: Supplementary file 1 — Supplementary Information [file 41467_2020_15539_MOESM1_ESM.pdf]

Supplementary Information

"Mild proteasomal stress improves photosynthetic performance in Arabidopsis chloroplasts"

by Grimmer et al.

Supplementary information

Supplementary Figure 1

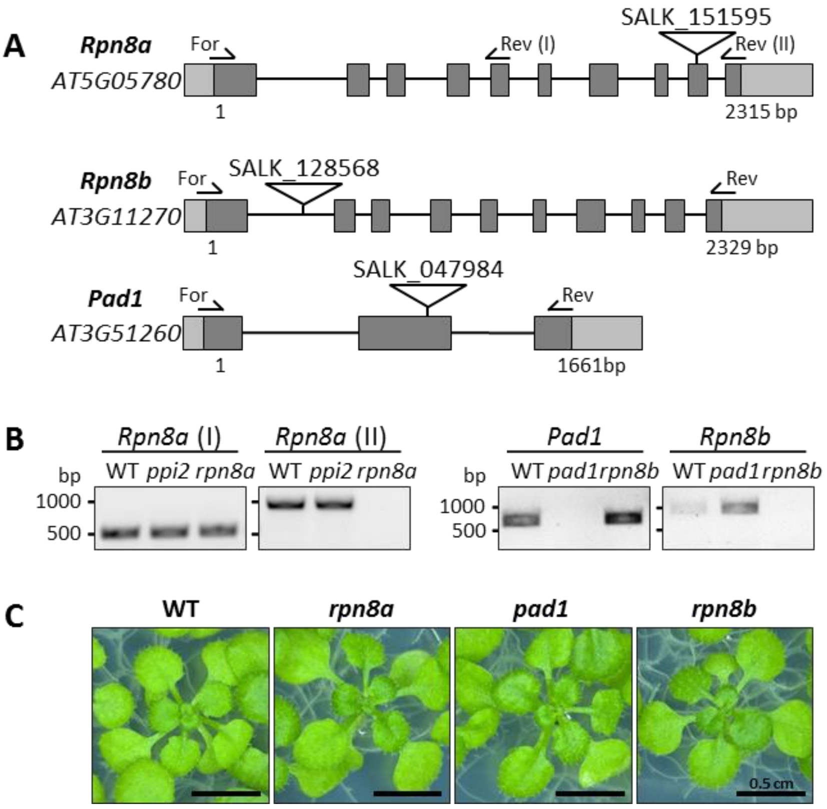

Suppl. Fig. 1: Characterization of proteasome mutants. (A) Location of the T-DNA insertion. (B) Transcript analysis. (C) Phenotype of the indicated single mutants.

Supplementary Figure 2

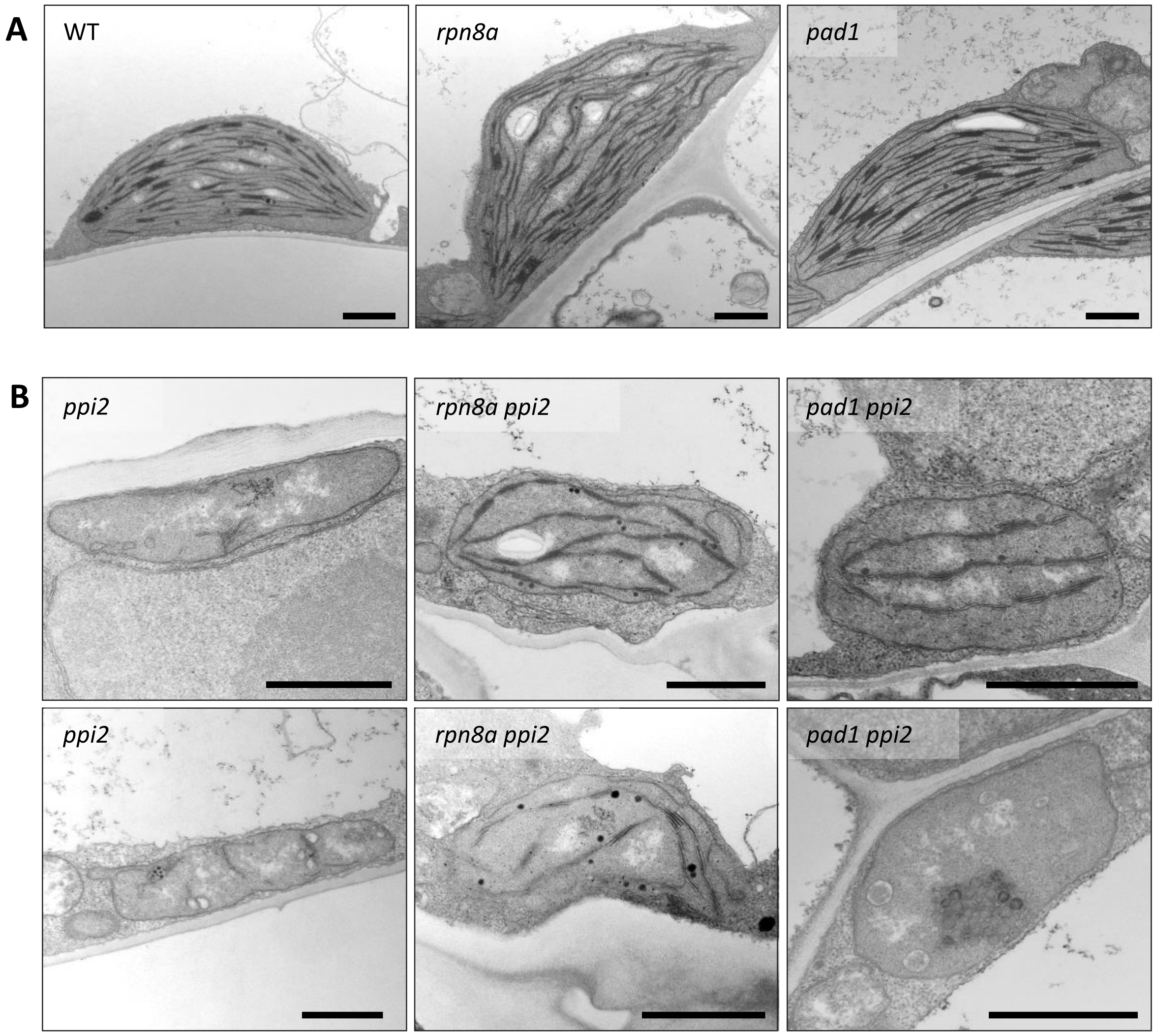

Suppl. Fig. 2: TEM-pictures of representative plastids from the indicated plant lines. (A) Wildtype and *rpn8a* and *pad1* single mutants, **(B)** *ppi2* and *rpn8a ppi2* and *pad1 ppi2* double mutants. The scale-bars represent 1 µm.

Supplementary Figure 3

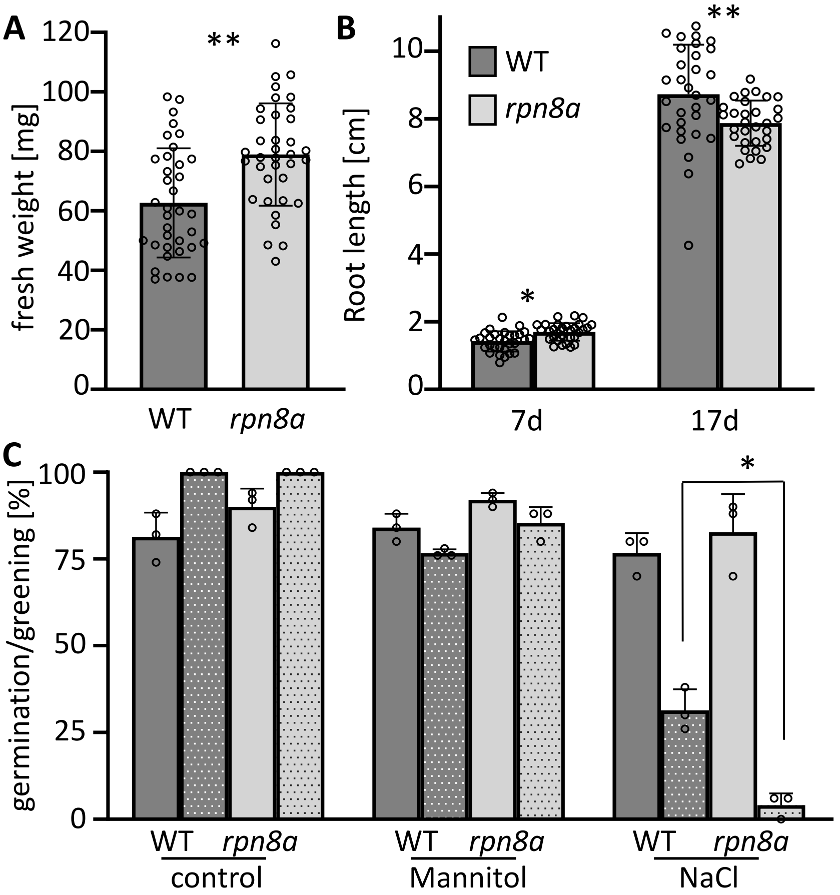

Suppl. Fig. 3: Growth of wildtype (WT) and *rpn8a* mutant plants. (A) Fresh-weight (n= 36) of four week old plants and (B) root-length (n= 30) determination of 7 and 17 days old wildtype and *rpn8a* mutant plants (B) Resilience against osmotic and salt stress of wildtype and *rpn8a* mutant plants (n= 150 (3 x 50 per pool)). The bars indicate the germination rate (%) of all seeds and the dotted bars the rate of green plants (of those germinated) as determined by visual inspection. Concentrations used were 250 mM Mannitol and 150 mM NaCl. Significant differences are indicated by one (p-value < 0.01) or two (p-value < 0.005) stars from a two-sided unpaired T-test.

<u>Supplementary Figure 4</u>

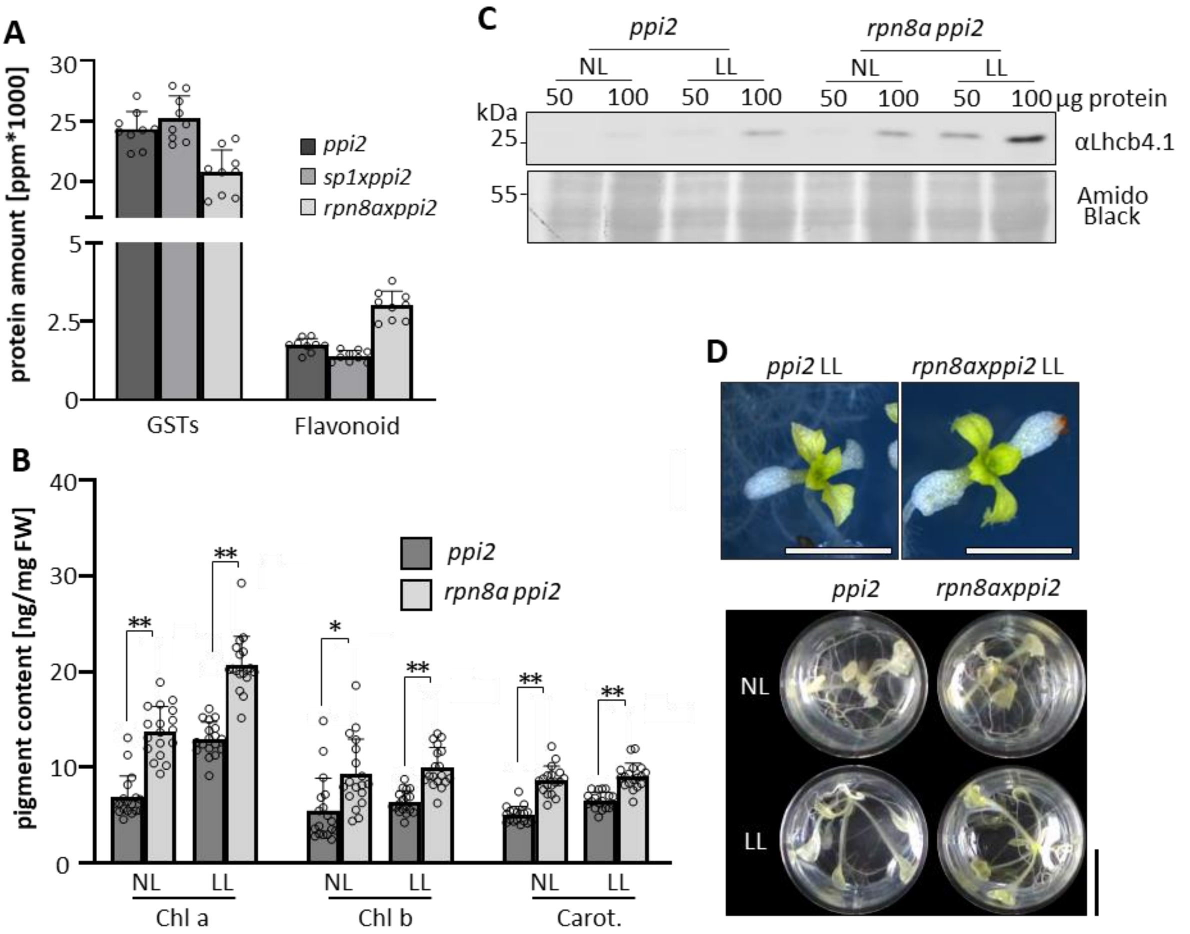

Suppl. Fig. 4: Influence of low-light conditions on plant growth and pigment accumulation. (A) Quantitative protein accumulation of the typical stress markers glutathione S-transferases (GSTs) and flavonoid biosynthetic enzymes reported as ppm abundance (n=9). (B) Pigment accumulation of 4-week old *ppi2* and *rpn8appi2* mutant plants after growth under normal light (NL, 150 µmol m-2 s-1) and under low light (LL, 20 µmol m-2 s-1) (n= 18). Significant differences are indicated by one (p-value > 0.005) or two (p-value > 0.0001) stars from a two-sided, unpaired T-test. (C) Immunoblotting results with the Lhcb4 antibody of the two mutants after application of 50µg and 100µg protein, as indicated. The amido black stain of the membrane is presented as a loading control. (D) Pictures of plants grown under NL or LL grown on plate (upper panel) or in liquid media in 96-well plates (lower panel). The upper scale bar represents, 0.5 cm, the lower scale bar represents 1 cm.

Supplementary Figure 5

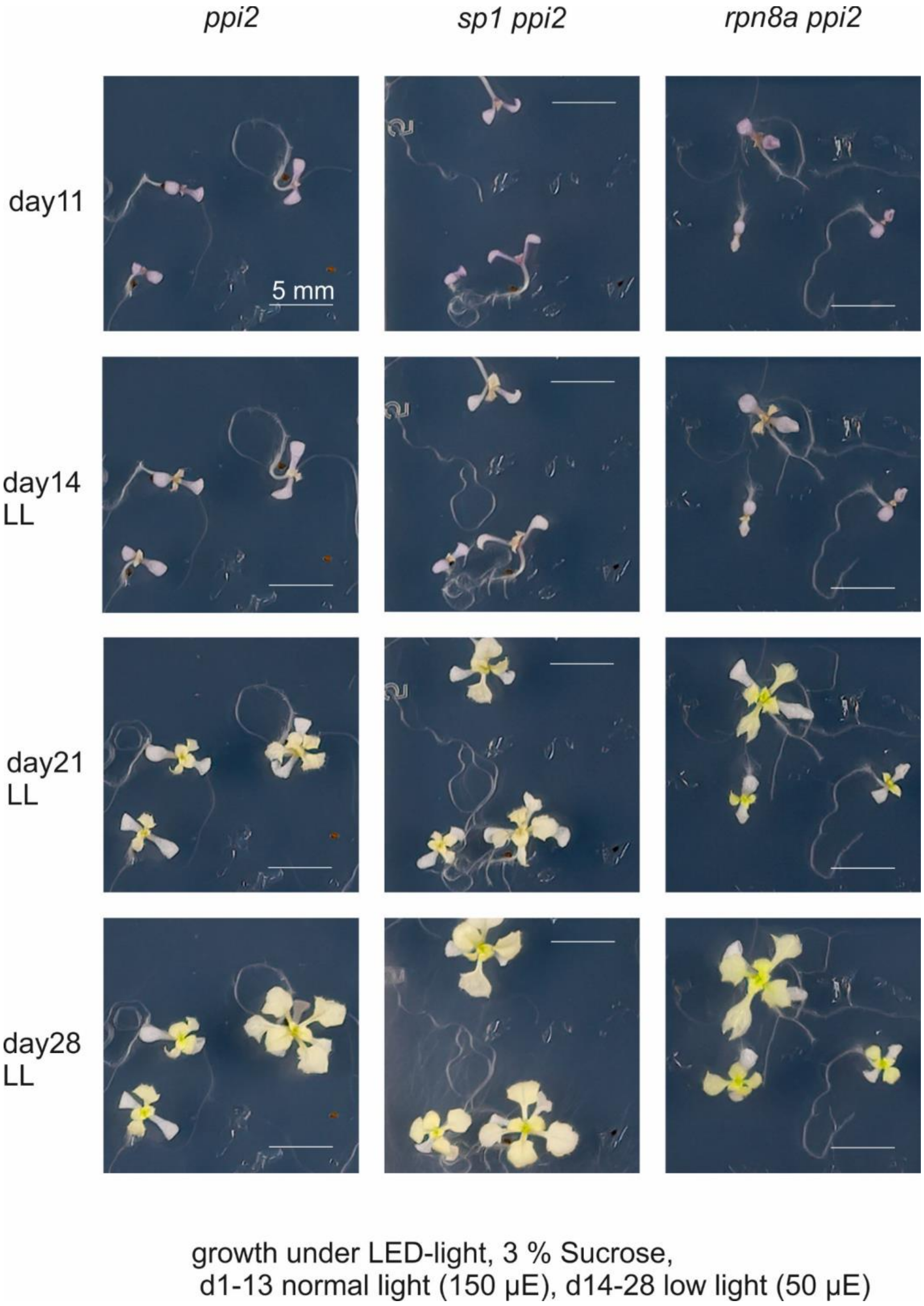

Suppl. Fig. 5: Comparison of growth of the three albino plant lines *ppi2*, *sp1 ppi2* and *rpn8a ppi2*. Plants were grown under 150 µE for 13 days and then shifted to lowlight conditions (50 µE). Representative plants are displayed. The scale bars represent 0.5 cm.

Supplementary Figure 6

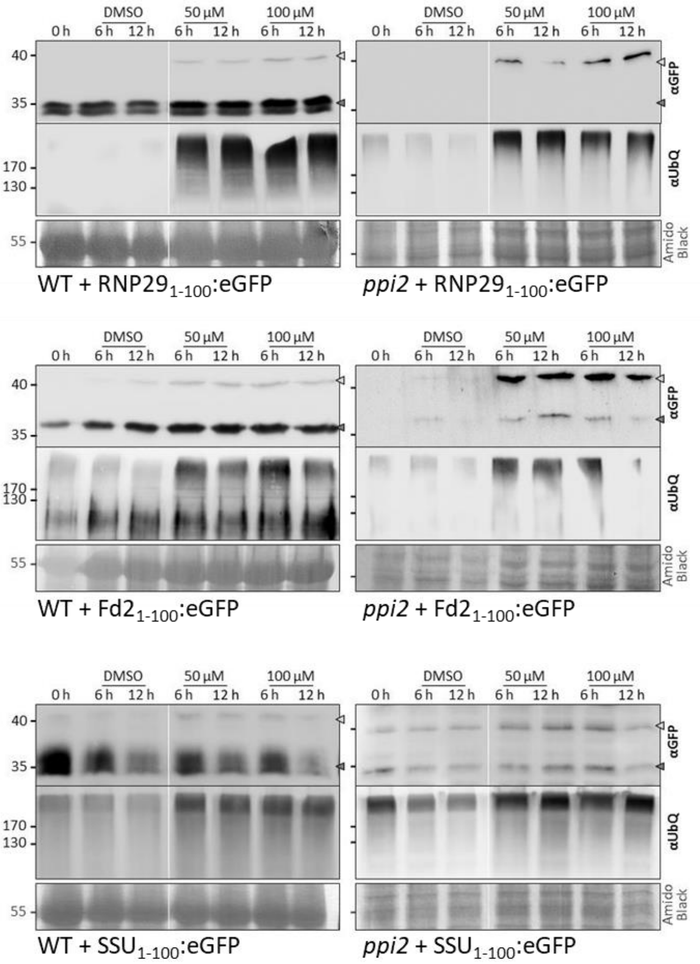

Suppl. Fig. 6: Influence of MG132 on precursor and mature protein accumulation in protoplast assays. Arabidopsis protoplasts were transformed with constructs encoding the first 100 amino acids of the indicated proteins fused to GFP. After transformation, the protoplasts were incubated with the indicated amount of MG132 for 6 or 12 hours. Proteins were extracted and subjected to western blotting using a GFP antibody for protein detection. The second panel shows the result of a ubiquitin antibody blot (αUbQ), the lower panel an excerpt of the amido black stain of the membrane. Representative results are shown, see also Fig. 4 B.
