# Supplementary material for: Mild proteasomal stress improves photosynthetic performance in Arabidopsis chloroplasts
Source: Nat Commun. 2020 Apr 3;11:1662. doi: 10.1038/s41467-020-15539-8 (PMC7125294; doi:10.1038/s41467-020-15539-8)
Supplement: Supplementary file 3 — Description of Additional Supplementary Files [file 41467_2020_15539_MOESM3_ESM.pdf]

Description of Additional Supplementary Files

File Name: Supplementary Data 1
Description: Quantitative proteomics data with wildtype (Col-0), sp1 and rpn8a plants, based on MSE protein quantification.

File Name: Supplementary Data 2
Description: Quantitative proteomics data with ppi2, sp1xppi2 and rpn8axppi2 plants, based on MSE protein quantification.

File Name: Supplementary Data 3
Description: Quantitative proteomics data with Col-0 (DMSO-control) and MG132-treated Col-0 plants, based on MSE protein quantification.

File Name: Supplementary Data 4
Description: Quantitative proteomics data with ppi2-DMSO control and ppi2-MG132-treated, based on MSE protein quantification.

File Name: Supplementary Data 5
Description: Identification of N-terminal peptides by CHAFRADIC in combination with iTRAQ quantification.

File Name: Supplementary Data 6
Description: Primer used for mutant characterization and real-time PCR analyses.

File Name: Supplementary Data 7
Description: Samle key for the datasets uploaded to PRIDE.
