# Supplementary material for: Mild proteasomal stress improves photosynthetic performance in Arabidopsis chloroplasts
Source: Nat Commun. 2020 Apr 3;11:1662. doi: 10.1038/s41467-020-15539-8 (PMC7125294; doi:10.1038/s41467-020-15539-8)
Supplement: Supplementary file 11 — Reporting Summary [file 41467_2020_15539_MOESM11_ESM.pdf]

nature research

Corresponding author(s): Sacha Baginsky
Last updated by author(s): March 08, 2020

Reporting Summary

Nature Research wishes to improve the reproducibility of the work that we publish. This form provides structure for consistency and transparency in reporting. For further information on Nature Research policies, see Authors & Referees and the Editorial Policy Checklist .

Statistics

For all statistical analyses, confirm that the following items are present in the figure legend, table legend, main text, or Methods section.

n/a	Confirmed	
☐	☒	The exact sample size (*n*) for each experimental group/condition, given as a discrete number and unit of measurement
☐	☒	A statement on whether measurements were taken from distinct samples or whether the same sample was measured repeatedly
☐	☒	The statistical test(s) used AND whether they are one- or two-sided<br>*Only common tests should be described solely by name; describe more complex techniques in the Methods section.*
☒	☐	A description of all covariates tested
☒	☐	A description of any assumptions or corrections, such as tests of normality and adjustment for multiple comparisons
☒	☐	A full description of the statistical parameters including central tendency (e.g. means) or other basic estimates (e.g. regression coefficient) AND variation (e.g. standard deviation) or associated estimates of uncertainty (e.g. confidence intervals)
☐	☒	For null hypothesis testing, the test statistic (e.g. *F*, *t*, *r*) with confidence intervals, effect sizes, degrees of freedom and *P* value noted<br>*Give P values as exact values whenever suitable.*
☒	☐	For Bayesian analysis, information on the choice of priors and Markov chain Monte Carlo settings
☒	☐	For hierarchical and complex designs, identification of the appropriate level for tests and full reporting of outcomes
☒	☐	Estimates of effect sizes (e.g. Cohen's *d*, Pearson's *r*), indicating how they were calculated

Our web collection on statistics for biologists contains articles on many of the points above.

Software and code

Policy information about availability of computer code

Data collection	AxioVision 4.8.2 (Carl Zeiss Microscopy), Image J (https://imagej.nih.gov/ij/), ImageJ Plugins NeuronJ (https://imagescience.org/meijering/soft ware/neuronj/), ImagingWin (http://www.walz.com/products/chl_p7 00/imaging-pam_ms/imagingwin.html), LSM Image Browser (Carl Zeiss Microscopy)
Data analysis	ProteinLynx Global Server (PLGS 3.0, Waters) A. thaliana protein data base TAIR10 (ftp://ftp.arabidopsis.org), MapMan (Thimm et al. , 2004), SUBA3 MS (Hooper et al. , 2014; Tanz et al. , 2013), data base of common contaminations (ftp://ftp.thegpm.org/fasta/cRAP/crap.fasta)

For manuscripts utilizing custom algorithms or software that are central to the research but not yet described in published literature, software must be made available to editors/reviewers. We strongly encourage code deposition in a community repository (e.g. GitHub). See the Nature Research guidelines for submitting code & software for further information.

Data

Policy information about availability of data

All manuscripts must include a data availability statement. This statement should provide the following information, where applicable:
- Accession codes, unique identifiers, or web links for publicly available datasets
- A list of figures that have associated raw data
- A description of any restrictions on data availability

All MS data were uploaded to PRIDE (https://www.ebi.ac.uk/pride) and are accessible via the identifier PXD014531 (single and double mutants), PXD014560 (MG132 dataset) and PXD017126 (ChaFRADIC dataset). A sample key for the uploaded data files with explanation is available in Supplementary Table 7. All other data are available in the text or in the supplement and in the source data file. All mutants and double mutants generate within the course of this MS are available upon request to the corresponding author.

Field-specific reporting

Please select the one below that is the best fit for your research. If you are not sure, read the appropriate sections before making your selection.

- [x] Life sciences
- [ ] Behavioural & social sciences
- [ ] Ecological, evolutionary & environmental sciences

For a reference copy of the document with all sections, see nature.com/documents/nr-reporting-summary-flat.pdf

Life sciences study design

All studies must disclose on these points even when the disclosure is negative.

Sample size	No sample size calculation was performed. We processed as many samples as reasonably possible.
Data exclusions	Data were excluded from the pigment, fresh weight and PAM measurements to equalize the sample size of each biological replicate. Selection of excluded data was randomized.
Replication	All experiments were repeated at least three times with independently grown plants, i.e. true biological replicates. The proper number of technical replicates or plants per measurement are provided in the figure legends.<br>Exception: Plants used for electron microscopy were grown once, but different plants were used for the replicates.
Randomization	Processing of the samples was randomized.<br>In MS measurements biological replicates were grouped. Technical replicates within the group were measured in a certain order with at least one blank after each measurement.
Blinding	Experiments were carried out by a single person

Reporting for specific materials, systems and methods

We require information from authors about some types of materials, experimental systems and methods used in many studies. Here, indicate whether each material, system or method listed is relevant to your study. If you are not sure if a list item applies to your research, read the appropriate section before selecting a response.

Materials & experimental systems

n/a	Involved in the study	
[ ]	[x]	Antibodies
[x]	[ ]	Eukaryotic cell lines
[x]	[ ]	Palaeontology
[ ]	[x]	Animals and other organisms
[x]	[ ]	Human research participants
[x]	[ ]	Clinical data

Methods

n/a	Involved in the study	
[x]	[ ]	ChIP-seq
[x]	[ ]	Flow cytometry
[x]	[ ]	MRI-based neuroimaging

Antibodies

Antibodies used	primary antibodies:<br>αGFP 598 (MBL International Corporation; 1:5000), αLhcb4 (AS04 045, Agrisera; 1:7000), αOEC33 (provided by R.B. Klösgen [Martin Luther University Halle-Wittenberg, Department of Plant Physiology]; 1:5000), αToc132 (provided by D. J. Schnell [Michigan State University, Department of Plant Biology]; 1:2000), αToc75 (Hiltbrunner et al., 2001; 1:2000), αUbQ11 (AS08 307, Agrisera; 1:10000)<br>secundary antibody: Goat anti-Rabbit (H&L) (AS09 602, Agrisera; 1:10000)
Validation	αGFP 598 (https://www.mblintl.com/products/598)<br>αLhcb4 (https://www.agrisera.com/en/artiklar/lhcb4-cp29-chlorophyll-a_b-binding-protein-of-plant-psii-.html)<br>αOEC33 (P. sativum, A. thaliana)<br>αToc132 (A. thaliana)<br>αToc75 (Hiltbrunner et al., 2001)<br>αUbQ11 (https://www.agrisera.com/en/artiklar/ubiquitin-.html)

Animals and other organisms

Policy information about studies involving animals; ARRIVE guidelines recommended for reporting animal research

Laboratory animals	No (laboratory) animals were used in this studies.<br>Whole plants were grown under controlled growth conditions (see methods) in growth cabinets (CU-36L3, CLF Plant Climatics GmbH); the following plant lines were used for experiments:<br>wild type: Arabidopsis thaliana Columbia (Col-0)<br>A. thaliana T-DNA insertion lines: plastid protein import mutant 2 (ppi2, Introregression of CS11072 in the eyotype Columbia, Kubis et al. (2004)) provided by Paul Jarvis (University of Oxford, Department of Plant Sciences), rpn8a-2 (SALK_151595c, The Nottingham Arabidopsis Stock Centre (NASC)), sp1-2 (SALK_063571, The Nottingham Arabidopsis Stock Centre (NASC)), pad1 (SALK_047984C in the Col-background), double mutants rpn8axppi2, pad1xppi2 and sp1xppi2 generated by crossing the mentioned T-DNA lines
Wild animals	The study did not involve wild animals.
Field-collected samples	The study did not involve samples collected from the field.
Ethics oversight	No ethical approval or guidance was required while working with plants.

Note that full information on the approval of the study protocol must also be provided in the manuscript.
