# Supplementary material for: Mild proteasomal stress improves photosynthetic performance in Arabidopsis chloroplasts
Source: Nat Commun. 2020 Apr 3;11:1662. doi: 10.1038/s41467-020-15539-8 (PMC7125294; doi:10.1038/s41467-020-15539-8)
Supplement: Supplementary file 12 — Source Data [file 41467_2020_15539_MOESM12_ESM.pdf]

Source data file: „Mild proteasomal stress improves photosynthetic performance in Arabidopsis chloroplasts“

To Fig. 1: Documentation of plant growth and pigment content for the different genotypes characterized in this manuscript: Slide 1 growth of sp1, rpn8a, rpn8b and pad1 single mutants; Slide 2 growth of sp1 ppi2, rpn8a ppi2, rpn8b ppi2 and pad1 ppi2 double mutants. Representative plants are shown; Slide 3 pigment measurements, conducted with 18 (3 x 6) plants in sp1, rpn8a, rpn8b and pad1 single mutants and 90 plants for the sp1 ppi2, rpn8a ppi2, rpn8b ppi2 and pad1 ppi2 double mutants (every datapoint represents the measurement with 5 plants)

To Fig. 2: Slide 4 Thylakoid layer count for three thylakoid stacks per plastid, 53 plastids x 3 replicates, were analyzed for every genotype

To Fig. 3: Slide 5-Slide 8 PAM measurements for every indicated genotype. We derived PSII quantum yield, non photochemical quenching (NPQ) and electron transport rate (ETR) from the chlorophyll fluorescence measurements (represented in Figure 3)

To Fig. 4: Slide 9-Slide 14 Original uncropped antibody blots representing the cropped images in Figure 4

To Fig. 5: Slide 15 Real time PCR data represented in Figure 5 C derived for plastid encoded genes psaJ, psbA, psbN, ndhF, rbcL and the nuclear encoded genes RbcS, Lhcb4, Lhcb5, zeta carotene desaturase (ZDS)

To Fig. 6: Slide 16-Slide 18 Proteasome activity measurements represented in Figure 6 B, Slide 19 hypocotyl length measurements in the presence of MG132 represented in Figure 6 C; Slide 20 Real time PCR expression data represented in Figure 5 C for genes of the proteasomal lid (RPN8b, RPN5a), base (RPN10, RPT2a), 20S core (PAA2) and regulatory factors (CDC48a, NAS6) in rpn8a and rpn8a ppi2 mutants compared to their reference (wildtype and ppi2)

To Fig. 7: Slide 21 Original uncropped antibody blots representing the cropped images in Figure 7 A

To Supplementary Fig. 3: Slide 22 Determined fresh weight [mg], Root length [cm] and germination and greening efficiency under salt stress for *rpn8a* and WT

To Supplementary Fig. 4: Slide 23 Pigment measurements, conducted with 90 plants in ppi2 and rpn8a ppi2 double mutants (every datapoint represents the measurement with 5 plants) grown under Normal light conditions (NL, 150 µmol m-2 s-1) or Low Light conditions (LL, 20 µmol m-2 s-1)

Fig.01

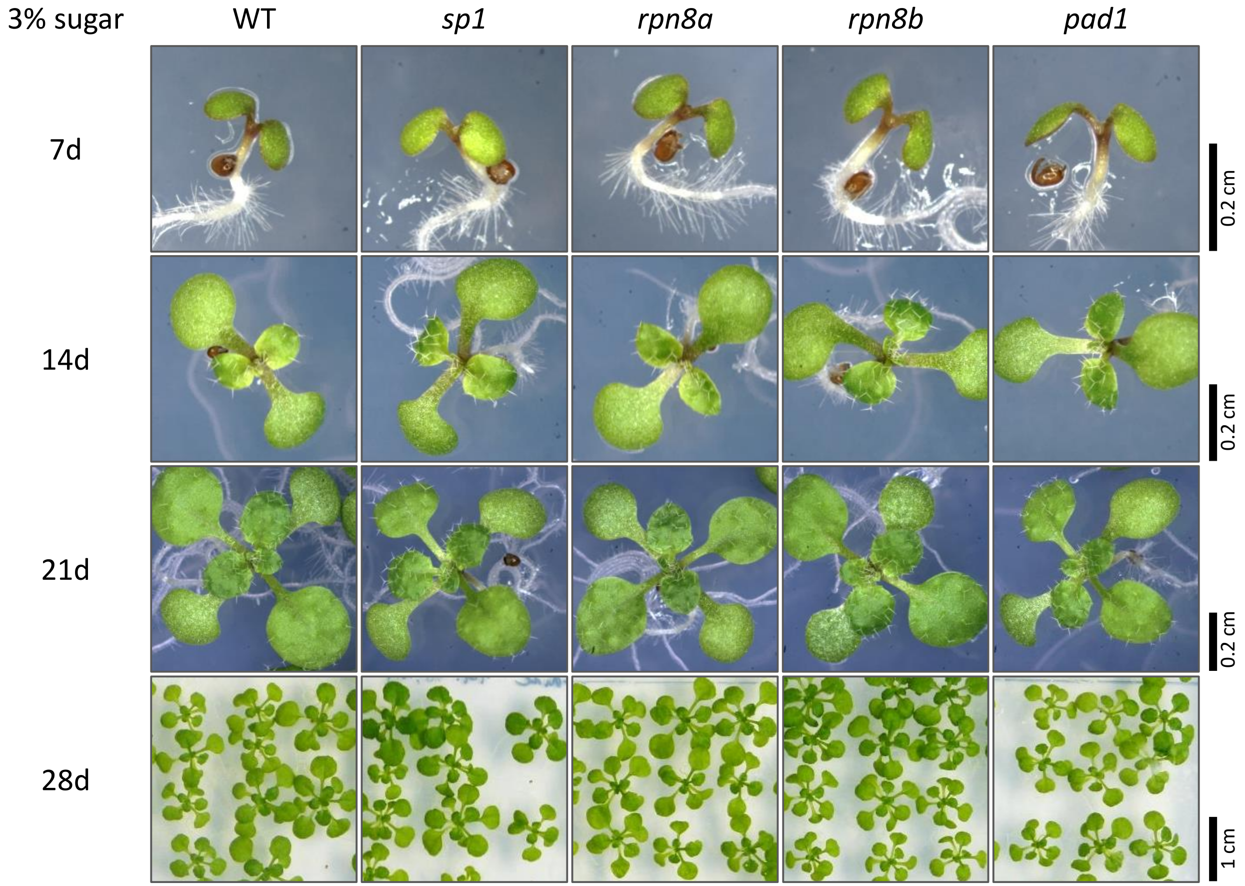
3% sugar
WT
sp1
rpn8a
rpn8b
pad1
7d
14d
21d
28d
0.2 cm
0.2 cm
0.2 cm
1 cm

Fig.01

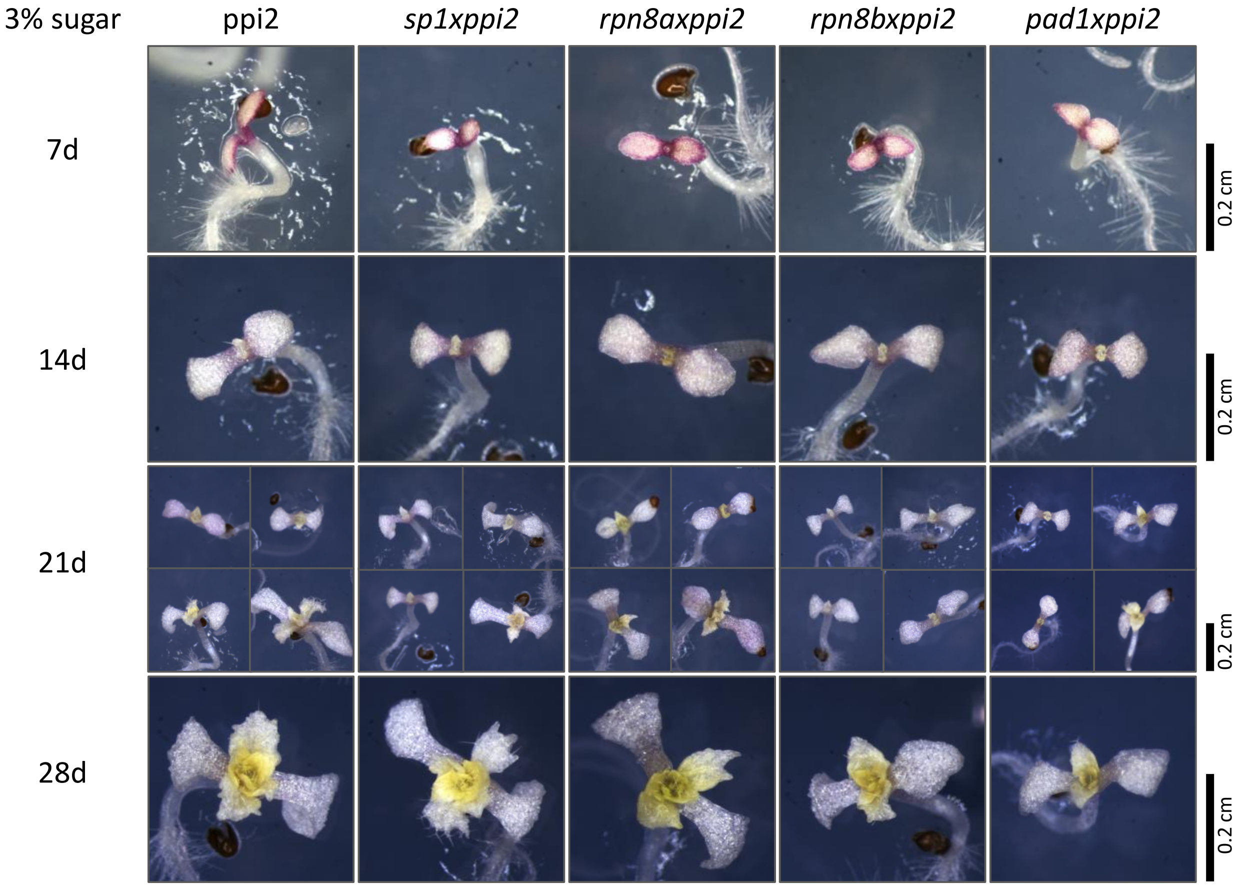

3% sugar
ppi2
sp1xppi2
rpn8axppi2
rpn8bxppi2
pad1xppi2
7d
14d
21d
28d
0.2 cm
0.2 cm
0.2 cm
0.2 cm

Fig.01

Values of pigment content [ng pigment/mg FW] (Biological replicate 1-3, n=6 each)

WT			*rpn8a_2*			*rpn8b*			*pad1*			*ppi2*			*rpn8axppi2*			*rpn8bxppi2*			*pad1xppi2*		
**Chl a**	**Chl b**	**Carot**	**Chl a**	**Chl b**	**Carot**	**Chl a**	**Chl b**	**Carot**	**Chl a**	**Chl b**	**Carot**	**Chl a**	**Chl b**	**Carot**	**Chl a**	**Chl b**	**Carot**	**Chl a**	**Chl b**	**Carot**	**Chl a**	**Chl b**	**Carot**
232.62	82.49	65.47	219.74	78.14	55.27	180.12	60.60	52.23	172.56	60.88	64.59	3.05	1.36	4.00	6.13	2.20	6.03	4.46	1.70	6.30	4.00	1.34	5.26
167.73	55.06	41.79	228.81	79.14	50.27	271.41	94.45	66.45	182.19	65.83	46.15	3.04	1.34	4.52	6.04	2.25	6.12	3.73	1.23	5.52	5.84	2.03	7.57
235.73	82.84	59.27	222.20	75.93	55.25	145.29	50.56	43.56	152.09	54.32	59.69	3.19	1.15	4.79	7.57	2.79	7.28	3.53	2.21	4.98	4.95	1.95	5.98
174.73	58.77	38.19	291.58	108.02	79.41	223.19	87.67	58.15	166.14	56.82	65.79	3.58	1.17	5.11	9.03	3.33	8.27	3.40	1.34	5.18	5.10	1.94	6.75
303.62	111.66	80.10	214.20	72.16	54.55	195.46	74.77	54.73	305.59	123.01	103.36	4.21	2.07	5.03	6.59	2.52	6.59	3.35	1.49	4.74	6.55	2.60	9.06
175.63	62.92	42.00	140.40	45.34	38.59	193.82	67.26	70.63	159.42	58.09	61.61	4.28	1.59	5.87	7.61	2.74	7.12	4.04	1.76	5.39	5.36	2.23	6.69
299.86	106.94	82.65	186.63	61.64	63.90	159.99	45.55	55.02	223.09	74.06	78.67	3.59	1.87	5.33	7.41	3.35	6.98	3.32	1.73	4.84	6.33	2.79	7.80
159.51	49.07	45.72	166.20	46.81	54.87	193.25	58.40	60.23	261.97	90.33	75.84	4.15	2.00	5.37	7.25	3.50	7.13	4.25	2.21	5.62	5.18	2.70	6.76
189.79	58.57	56.67	270.76	93.30	54.53	179.81	59.61	45.10	210.71	67.39	66.94	4.92	2.21	5.96	9.42	4.05	7.96	4.39	2.58	5.48	5.06	2.75	5.56
232.92	79.48	58.08	185.39	56.55	55.67	174.10	63.49	43.10	245.69	78.64	85.55	5.20	3.67	6.14	8.57	4.73	7.48	4.90	3.55	5.63	6.62	4.27	7.12
203.18	63.57	53.87	221.35	75.48	63.38	196.02	62.60	57.34	156.99	51.62	47.31	4.37	3.67	5.00	9.47	5.07	8.36	4.82	3.50	5.54	4.86	2.65	5.61
152.80	44.51	51.08	198.84	62.01	47.39	275.21	95.87	72.19	220.84	72.89	60.28	4.81	2.95	5.81	8.11	4.72	6.86	5.45	4.02	6.62	7.19	4.55	8.24
180.65	62.62	37.93	152.54	44.66	45.54	334.82	114.80	60.02	224.38	74.27	71.32	2.99	1.55	5.49	7.50	3.62	8.30	2.90	1.70	5.88	3.80	4.76	6.78
230.57	81.65	49.14	170.90	54.61	33.74	263.84	97.79	51.94	213.71	72.94	39.42	3.86	2.01	5.01	5.29	3.00	7.54	4.04	2.23	5.64	3.73	2.07	6.38
261.77	99.18	44.41	219.10	77.52	43.62	301.86	114.93	58.09	362.44	136.66	107.84	3.69	2.26	5.85	6.18	3.33	6.65	3.84	2.00	5.80	3.19	2.30	6.87
226.25	88.09	40.18	206.83	78.31	39.85	169.73	59.18	39.31	233.89	84.52	60.83	2.56	1.77	5.36	7.67	3.75	7.57	4.17	2.29	6.35	4.69	2.44	6.92
174.99	65.53	35.23	323.56	127.14	52.07	127.14	50.97	29.50	180.51	63.31	34.86	*2.49*	*2.06*	*4.57*	*7.52*	*3.79*	7.67	*3.09*	*2.58*	*5.85*	*3.71*	*2.04*	*6.17*
258.25	106.48	50.28	191.36	68.80	38.10	319.65	124.80	75.65	293.22	111.54	71.10	3.39	2.41	5.43	5.95	2.87	6.24	3.72	2.58	6.09	3.25	2.58	6.38
**p Value [WT vs. mut]**			0.858	0.673	0.935	0.894	0.863	0.433	0.740	0.781	0.011	**p Value [ppi2 vs. alb mut]**			0.000	0.000	0.000	0.367	0.438	0.042	0.001	0.039	0.000

WT			*sp1-2*			*rpn8a_2*			*ppi2*			*ppi2xsp1-2*			*ppi2xrpn8a_2*		
**Chl a**	**Chl b**	**Carot**	**Chl a**	**Chl b**	**Carot**	**Chl a**	**Chl b**	**Carot**	**Chl a**	**Chl b**	**Carot**	**Chl a**	**Chl b**	**Carot**	**Chl a**	**Chl b**	**Carot**
211.97	79.76	42.40	213.89	67.49	52.23	180.75	62.19	41.13	5.49	2.50	5.21	6.41	2.73	7.08	10.21	4.40	7.22
144.93	48.86	30.51	180.48	62.50	50.44	136.29	44.98	28.94	5.23	3.46	4.01	7.85	5.54	7.39	13.68	7.39	10.16
217.66	82.34	45.45	126.27	40.63	27.79	138.50	45.36	30.86	6.21	3.07	5.93	13.90	10.17	8.41	11.29	6.31	8.19
207.15	72.33	51.21	214.24	75.10	46.46	131.53	43.55	32.24	5.49	2.97	4.60	7.39	4.35	6.84	13.11	7.03	9.26
143.40	52.11	34.98	243.48	93.36	54.38	190.17	68.06	48.65	5.51	2.98	4.52	7.18	3.11	7.21	16.41	8.17	12.21
143.12	52.38	39.15	138.71	48.14	31.81	158.41	54.53	49.92	6.26	3.54	5.44	6.34	3.14	6.56	9.35	4.71	6.71
149.85	49.70	36.96	157.79	58.65	39.12	177.55	61.36	43.76	6.76	4.39	5.21	4.12	2.92	4.08	11.98	5.60	8.84
113.82	36.27	30.88	152.61	51.86	39.59	183.18	63.95	45.51	11.33	11.73	5.30	7.88	7.15	5.49	14.57	9.13	9.15
125.22	41.87	31.56	216.50	80.92	57.38	215.52	76.14	53.73	4.57	2.82	4.18	4.75	3.24	4.59	14.95	11.94	7.68
123.61	39.01	39.05	177.61	59.14	47.13	177.39	60.19	47.08	6.50	6.88	4.41	6.46	5.38	4.90	10.46	7.95	6.08
147.01	47.61	42.65	164.07	60.02	34.48	156.17	68.18	38.84	5.69	3.60	4.40	6.92	4.94	6.41	14.54	7.97	10.91
154.20	49.93	36.80	233.07	84.04	58.78	305.89	109.96	69.44	5.41	3.89	4.33	8.98	9.50	5.65	12.58	8.53	8.15
118.32	46.80	26.96	217.95	74.12	54.70	191.83	76.12	40.93	13.11	14.88	7.49	15.52	19.45	6.61	18.93	18.58	8.53
169.00	94.47	34.16	153.26	52.33	31.69	140.08	46.30	34.58	6.68	5.00	5.52	7.34	7.25	3.91	13.97	8.30	8.63
141.93	57.21	37.28	154.11	60.42	32.41	161.26	55.31	33.25	6.19	5.50	4.26	6.70	6.44	4.60	16.60	13.60	8.45
261.53	94.83	81.30	190.11	61.54	45.51	233.87	76.09	53.11	8.20	7.18	5.07	8.81	8.97	5.72	17.04	14.17	9.48
149.13	50.61	40.36	189.55	64.64	42.15	289.85	104.46	59.61	8.95	8.95	6.15	7.20	6.77	5.87	16.00	12.90	8.89
184.71	64.46	48.84	176.98	60.99	39.27	162.73	61.70	39.75	6.84	5.83	5.36	9.93	9.79	6.49	12.56	10.48	7.20
**p Value [WT vs. mut]**			0.085	0.325	0.408	0.124	0.292	0.378	**p Value [ppi2 vs. alb mut]**			0.214	0.339	0.015	0.000	0.003	0.000

Fig.02

#Thylakoid layers in albino mutants (3 slices, n=51 plastids à 3 thylakoid stacks each)

	ppi2									*rpn8a ppi2*									*pad1 ppi2*								
Plastid #	1-1	1-2	1-3	2-1	2-2	2-3	3-1	3-2	3-3	1-1	1-2	1-3	2-1	2-2	2-3	3-1	3-2	3-3	1-1	1-2	1-3	2-1	2-2	2-3	3-1	3-2	3-3
1	3	1	1	4	1	1	2	2	2	3	3	1	2	2	1	3	2	1	3	2	2	2	2	1	1	2	1
2	1	2	2	2	2	2	2	1	1	3	2	3	4	2	1	4	4	2	3	3	2	2	1	1	2	2	1
3	1	1	3	3	2	2	1	1	1	2	2	2	3	3	1	2	2	1	2	2	2	1	1	1	1	1	1
4	2	2	2	3	2	1	1	1	1	2	2	2	4	4	3	2	1	1	3	2	2	1	1	1	2	1	1
5	2	2	1	3	2	2	2	1	1	2	2	1	4	4	4	3	3	3	2	2	1	1	1	1	2	2	1
6	2	2	1	1	1	1	1	1	1	2	2	2	3	3	2	2	2	1	2	1	1	2	1	1	2	2	1
7	3	2	2	1	1	1	2	2	1	1	1	1	4	2	2	2	1	1	2	1	1	2	1	1	2	2	1
8	3	2	1	2	2	2	1	1	1	2	2	1	5	2	1	3	2	2	2	2	2	1	1	1	2	1	1
9	2	1	1	2	2	2	2	1	1	2	2	1	7	2	1	3	3	2	2	2	2	2	2	1	2	2	2
10	2	2	2	3	2	2	2	1	1	2	2	2	3	3	1	1	1	1	2	1	1	2	1	1	2	2	1
11	2	2	1	2	2	2	2	2	1	3	2	1	5	4	3	3	2	1	2	2	2	1	1	1	2	1	1
12	3	3	2	2	2	2	3	2	2	2	2	2	5	5	4	3	2	1	4	2	2	2	1	1	2	2	2
13	1	1	2	2	2	2	2	2	1	2	1	1	4	4	3	3	3	2	2	2	2	1	1	1	2	1	1
14	2	2	1	2	2	2	2	2	2	2	1	1	4	4	3	1	1	1	2	1	1	2	2	1	3	2	1
15	2	2	1	1	1	1	1	1	1	2	2	2	4	4	2	2	1	1	2	1	1	2	2	2	2	2	1
16	2	2	2	2	2	1	2	1	1	2	1	1	2	2	2	2	1	1	2	2	2	2	1	1	2	1	2
17	1	1	1	2	2	2	2	1	1	3	3	2	4	2	2	3	3	1	2	2	2	2	1	1	2	2	1
18	2	1	1	1	1	1	1	1	1	3	2	1	4	4	2	3	3	1	2	2	2	1	1	1	2	2	1
19	2	2	1	2	2	1	1	1	1	4	3	3	4	3	2	3	3	3	4	3	3	2	1	1	2	2	1
20	3	2	2	3	3	3	2	1	1	2	2	2	5	5	3	2	1	1	2	2	2	2	1	1	2	2	1
21	1	1	1	2	2	2	2	2	2	3	3	2	3	4	1	2	2	1	2	1	1	2	2	1	1	1	1
22	1	1	1	3	3	1	1	1	1	2	2	2	3	3	2	3	2	2	2	1	1	2	2	1	2	2	1
23	1	1	1	4	2	1	2	1	1	2	2	1	2	2	1	2	2	1	1	1	1	2	1	1	2	2	1
24	1	2	1	3	2	2	3	2	1	2	2	1	4	1	1	3	3	2	2	2	2	1	1	1	2	1	1
25	1	2	1	1	1	1	1	1	1	2	2	1	5	4	4	4	2	2	3	2	2	1	1	1	2	2	1
26	1	1	1	2	2	2	2	2	2	4	2	1	5	4	3	2	2	2	2	1	1	1	1	1	2	3	1
27	2	1	1	2	2	1	2	1	1	2	1	1	3	1	1	2	2	2	2	2	2	2	2	2	2	2	1
28	1	1	1	1	1	1	1	1	1	4	4	4	2	2	1	2	2	1	2	2	2	2	1	1	2	2	2
29	2	2	1	1	1	1	1	1	1	3	3	2	1	1	1	5	4	3	3	2	2	1	1	1	3	2	2
30	2	2	2	2	1	1	2	2	1	4	3	3	3	2	1	1	1	1	2	2	2	2	1	1	1	1	1
31	1	1	1	3	3	2	1	1	1	4	3	2	6	4	2	4	3	2	2	2	2	2	2	1	2	2	1
32	1	1	1	3	1	1	2	2	1	3	3	1	5	4	3	2	2	1	2	2	2	1	1	1	2	2	1
33	1	2	1	2	2	1	1	1	1	4	2	1	3	3	2	3	2	1	2	1	1	1	1	1	2	2	1
34	2	1	1	3	2	1	2	2	1	2	1	1	3	2	2	2	2	2	2	2	2	3	1	1	2	1	1
35	1	2	2	1	1	1	1	1	1	4	3	2	3	3	2	2	2	1	2	2	2	1	1	1	3	2	2
36	2	2	1	1	1	1	1	1	1	3	3	3	3	1	1	3	2	2	3	2	2	1	1	1	1	1	1
37	2	2	2	2	2	1	1	1	1	3	3	2	3	1	2	2	2	1	2	2	2	1	1	1	2	2	2
38	1	1	1	2	1	1	2	1	1	3	3	3	2	2	1	3	2	2	2	2	2	1	1	1	2	2	1
39	2	2	1	1	1	1	1	1	1	3	2	1	5	5	4	4	2	2	3	2	2	3	1	1	1	2	1
40	2	1	1	2	1	1	1	1	1	4	4	2	2	2	1	2	2	2	2	2	2	1	1	1	2	2	1
41	2	1	1	2	1	1	2	1	1	4	4	3	3	3	1	5	4	3	2	2	2	1	1	1	2	1	1
42	2	1	1	1	1	1	2	2	1	3	3	2	3	4	3	4	1	1	2	1	1	1	1	1	2	1	1
43	1	1	1	1	1	1	2	1	1	3	3	3	2	1	1	4	3	2	2	2	2	1	1	1	1	2	2
44	2	2	2	2	1	1	2	2	2	3	1	1	3	1	1	2	2	1	2	2	2	2	1	1	2	2	1
45	2	1	1	1	1	1	2	2	1	3	3	3	5	3	1	3	2	1	3	2	2	1	1	1	3	2	2
46	2	1	1	2	2	1	2	2	1	2	1	1	1	1	1	2	1	1	2	2	2	1	1	1	2	2	1
47	1	1	1	2	1	1	2	1	1	4	3	1	4	3	2	2	1	1	1	1	1	1	1	1	2	1	1
48	1	1	1	3	2	3	1	1	1	3	3	2	3	1	1	4	4	1	2	2	2	1	1	1	2	2	2
49	1	1	1	2	2	2	2	2	2	3	3	2	3	2	1	4	3	2	2	2	2	1	1	1	3	1	1
50	2	1	1	2	1	1	2	2	1	3	1	1	2	2	1	5	3	3	2	2	2	2	2	1	2	2	2
51	1	1	1	3	2	2	2	2	2	3	2	2	3	3	1	2	1	1	2	2	2	1	1	1	1	2	1

Fig.03

PAM data – WT (Biological replicate 1-3, n=6 each)

PAR	Y(II)1	Y(II)2	Y(II)3	Y(II)4	Y(II)5	Y(II)6	Y(II)1	Y(II)2	Y(II)3	Y(II)4	Y(II)5	Y(II)6	Y(II)1	Y(II)2	Y(II)3	Y(II)4	Y(II)5	Y(II)6	MEAN	SEM
**0**	0.806	0.813	0.806	0.816	0.810	0.816	0.813	0.816	0.816	0.809	0.807	0.805	0.795	0.808	0.810	0.803	0.799	0.799	0.808	0.0015
**14**	0.446	0.446	0.474	0.463	0.436	0.471	0.581	0.532	0.504	0.474	0.447	0.451	0.539	0.505	0.489	0.470	0.444	0.463	0.480	0.0090
**33**	0.442	0.420	0.466	0.456	0.434	0.465	0.521	0.483	0.458	0.457	0.428	0.412	0.508	0.463	0.481	0.454	0.429	0.446	0.457	0.0066
**52**	0.406	0.399	0.440	0.419	0.404	0.420	0.490	0.451	0.431	0.421	0.394	0.380	0.484	0.433	0.449	0.434	0.411	0.420	0.427	0.0066
**73**	0.394	0.389	0.430	0.408	0.396	0.406	0.482	0.446	0.423	0.409	0.385	0.374	0.477	0.432	0.442	0.432	0.404	0.403	0.418	0.0068
**102**	0.377	0.372	0.407	0.394	0.379	0.395	0.470	0.431	0.407	0.390	0.371	0.362	0.466	0.421	0.441	0.412	0.405	0.402	0.406	0.0071
**132**	0.370	0.363	0.393	0.387	0.364	0.390	0.464	0.421	0.400	0.376	0.371	0.354	0.466	0.415	0.439	0.419	0.406	0.402	0.400	0.0075
**170**	0.359	0.356	0.378	0.375	0.359	0.374	0.454	0.403	0.390	0.366	0.348	0.340	0.458	0.416	0.425	0.411	0.395	0.389	0.389	0.0078
**217**	0.348	0.341	0.358	0.358	0.335	0.359	0.431	0.392	0.366	0.346	0.344	0.329	0.441	0.398	0.416	0.392	0.383	0.374	0.373	0.0076
**285**	0.319		0.323	0.327	0.307	0.329	0.403	0.371	0.336	0.320	0.323	0.304	0.414	0.379	0.388	0.364	0.357	0.357	0.348	0.0080
**360**	0.287	0.299	0.293	0.298	0.271	0.303	0.369	0.340	0.312	0.292	0.296	0.280	0.381	0.351	0.371	0.330	0.326	0.324	0.318	0.0076
**458**	0.257	0.261	0.254	0.266	0.235	0.268	0.327	0.312	0.279	0.266	0.263	0.243	0.338	0.314	0.339	0.292	0.293	0.296	0.284	0.0073

PAR	Y(NPQ)1	Y(NPQ)2	Y(NPQ)3	Y(NPQ)4	Y(NPQ)5	Y(NPQ)6	Y(NPQ)1	Y(NPQ)2	Y(NPQ)3	Y(NPQ)4	Y(NPQ)5	Y(NPQ)6	Y(NPQ)1	Y(NPQ)2	Y(NPQ)3	Y(NPQ)4	Y(NPQ)5	Y(NPQ)6	MEAN	SEM
**0**	0.006	0.005	0.006	0.005	0.006	0.005	0.006	0.006	0.005	0.006	0.006	0.006	0.006	0.006	0.006	0.006	0.006	0.006	0.006	0.0001
**14**	0.041	0.040	0.038	0.039	0.040	0.039	0.037	0.034	0.037	0.040	0.045	0.041	0.033	0.030	0.032	0.032	0.033	0.040	0.037	0.0009
**33**	0.182	0.190	0.147	0.178	0.180	0.185	0.187	0.184	0.174	0.206	0.213	0.208	0.132	0.182	0.145	0.150	0.151	0.177	0.176	0.0052
**52**	0.298	0.294	0.247	0.291	0.290	0.305	0.267	0.273	0.263	0.303	0.305	0.295	0.211	0.268	0.245	0.244	0.258	0.266	0.274	0.0060
**73**	0.332	0.328	0.281	0.318	0.314	0.340	0.283	0.294	0.289	0.333	0.336	0.328	0.235	0.283	0.269	0.271	0.291	0.306	0.302	0.0066
**102**	0.358	0.355	0.314	0.336	0.336	0.352	0.293	0.317	0.317	0.363	0.370	0.359	0.261	0.309	0.290	0.306	0.314	0.330	0.327	0.0068
**132**	0.366	0.366	0.329	0.341	0.348	0.354	0.296	0.331	0.334	0.382	0.383	0.377	0.270	0.323	0.303	0.316	0.321	0.342	0.338	0.0071
**170**	0.378	0.375	0.345	0.351	0.350	0.366	0.307	0.354	0.353	0.399	0.413	0.395	0.288	0.330	0.325	0.333	0.340	0.361	0.354	0.0073
**217**	0.390	0.389	0.365	0.365	0.366	0.373	0.330	0.369	0.381	0.421	0.425	0.412	0.311	0.350	0.342	0.357	0.360	0.382	0.372	0.0068
**285**	0.416		0.396	0.389	0.384	0.396	0.356	0.391	0.415	0.450	0.449	0.437	0.342	0.372	0.375	0.391	0.388	0.404	0.397	0.0070
**360**	0.443	0.432	0.419	0.411	0.410	0.415	0.388	0.419	0.440	0.478	0.477	0.461	0.376	0.396	0.395	0.425	0.421	0.436	0.425	0.0065
**458**	0.467	0.460	0.452	0.436	0.435	0.441	0.429	0.447	0.472	0.502	0.510	0.495	0.421	0.433	0.431	0.463	0.453	0.466	0.456	0.0059

PAR	ETR1	ETR2	ETR3	ETR4	ETR5	ETR6	ETR1	ETR2	ETR3	ETR4	ETR5	ETR6	ETR1	ETR2	ETR3	ETR4	ETR5	ETR6	MEAN	SEM
**0**	0.00	0.00	0.00	0.00	0.00	0.00	0.00	0.00	0.00	0.00	0.00	0.00	0.00	0.00	0.00	0.00	0.00	0.00	0.00	0.0000
**14**	2.60	2.60	2.80	2.70	2.60	2.80	4.10	3.70	3.50	3.30	3.10	3.20	3.80	3.50	3.40	3.30	3.10	3.20	3.18	0.1013
**33**	5.60	5.30	5.90	5.70	5.50	5.90	7.80	7.20	6.90	6.90	6.40	6.20	7.60	7.00	7.20	6.80	6.40	6.70	6.50	0.1694
**52**	8.00	7.90	8.70	8.30	8.00	8.30	11.50	10.60	10.10	9.90	9.30	8.90	11.40	10.20	10.60	10.20	9.70	9.90	9.53	0.2647
**73**	10.60	10.40	11.60	11.00	10.70	10.90	15.40	14.30	13.50	13.10	12.30	12.00	15.30	13.80	14.10	13.80	12.90	12.90	12.70	0.3667
**102**	14.20	14.10	15.40	14.90	14.30	14.90	21.20	19.40	18.30	17.60	16.70	16.30	21.00	18.90	19.80	18.60	18.20	18.10	17.33	0.5319
**132**	18.00	17.70	19.20	18.90	17.70	19.00	26.90	24.40	23.20	21.80	21.50	20.50	27.00	24.10	25.50	24.30	23.50	23.30	22.03	0.7116
**170**	22.60	22.40	23.80	23.60	22.60	23.60	34.00	30.20	29.20	27.50	26.10	25.50	34.30	31.20	31.90	30.80	29.60	29.10	27.67	0.9182
**217**	28.40	27.80	29.20	29.20	27.30	29.30	41.80	38.00	35.50	33.50	33.30	31.90	42.80	38.60	40.30	38.00	37.10	36.30	34.35	1.1575
**285**	34.30		34.70	35.20	33.00	35.40	51.50	47.50	43.10	41.00	41.40	39.00	53.00	48.50	49.60	46.60	45.80	45.70	42.66	1.5319
**360**	39.00	40.50	39.70	40.40	36.80	41.10	59.60	55.00	50.40	47.10	47.90	45.20	61.60	56.70	59.90	53.40	52.70	52.30	48.85	1.8375
**458**	44.20	44.90	43.60	45.70	40.30	46.10	67.00	63.80	57.00	54.40	53.70	49.70	69.20	64.20	69.30	59.70	59.90	60.40	55.17	2.1883

Fig.03

PAM data – *rpn8a* (Biological replicate 1-3, n=6 each)

PAR	Y(II)1	Y(II)2	Y(II)3	Y(II)4	Y(II)5	Y(II)6	Y(II)1	Y(II)2	Y(II)3	Y(II)4	Y(II)5	Y(II)6	Y(II)1	Y(II)2	Y(II)3	Y(II)4	Y(II)5	Y(II)6	MEAN	SEM	p Value *[rpn8a vs. WT]*
0	0.817	0.810	0.818	0.814	0.811	0.809	0.811	0.808	0.814	0.801	0.806	0.813	0.798	0.808	0.802	0.806	0.802	0.808	0.809	0.0013	0.803
14	0.472	0.510	0.482	0.502	0.464	0.477	0.570	0.539	0.509	0.508	0.531	0.506	0.565	0.509	0.523	0.505	0.495	0.524	0.511	0.0066	0.011
33	0.476	0.491	0.474	0.494	0.455	0.465	0.529	0.505	0.470	0.491	0.510	0.488	0.524	0.477	0.498	0.485	0.469	0.503	0.489	0.0046	0.000
52	0.438	0.460	0.436	0.465	0.412	0.437	0.498	0.472	0.440	0.463	0.477	0.464	0.510	0.452	0.481	0.462	0.448	0.486	0.461	0.0056	0.000
73	0.430	0.453	0.429	0.447	0.403	0.420	0.483	0.466	0.433	0.461	0.473	0.458	0.504	0.448	0.478	0.459	0.442	0.480	0.454	0.0058	0.001
102	0.416	0.448	0.417	0.434	0.385	0.413	0.475	0.450	0.417	0.455	0.467	0.454	0.495	0.435	0.480	0.449	0.437	0.480	0.445	0.0065	0.000
132	0.421	0.449	0.402	0.424	0.374	0.406	0.464	0.446	0.402	0.448	0.461	0.448	0.498	0.429	0.477	0.445	0.441	0.477	0.440	0.0071	0.001
170	0.405	0.435	0.390	0.406	0.357	0.389	0.448	0.431	0.394	0.437	0.448	0.439	0.485	0.425	0.465	0.437	0.427	0.474	0.427	0.0075	0.001
217	0.394	0.417	0.376	0.394	0.333	0.375	0.427	0.407	0.380	0.417	0.429	0.422	0.472	0.407	0.452	0.415	0.414	0.460	0.411	0.0076	0.002
285	0.367	0.385	0.349	0.361	0.302	0.334	0.392	0.379	0.352	0.378	0.397	0.393	0.441	0.374	0.410	0.386	0.388	0.434	0.379	0.0076	0.011
360	0.343	0.368	0.322	0.326	0.275	0.302	0.350	0.338	0.317	0.354	0.364	0.360	0.397	0.345	0.376	0.353	0.361	0.396	0.347	0.0071	0.010
458	0.296	0.308	0.290	0.282	0.244	0.258	0.312	0.309	0.281	0.311	0.326	0.316	0.354	0.307	0.331	0.307	0.319	0.347	0.305	0.0063	0.035

PAR	Y(NPQ)1	Y(NPQ)2	Y(NPQ)3	Y(NPQ)4	Y(NPQ)5	Y(NPQ)6	Y(NPQ)1	Y(NPQ)2	Y(NPQ)3	Y(NPQ)4	Y(NPQ)5	Y(NPQ)6	Y(NPQ)1	Y(NPQ)2	Y(NPQ)3	Y(NPQ)4	Y(NPQ)5	Y(NPQ)6	MEAN	SEM	p Value *[rpn8a vs. WT]*
0	0.006	0.006	0.005	0.005	0.006	0.006	0.006	0.006	0.006	0.006	0.006	0.006	0.006	0.006	0.006	0.006	0.006	0.006	0.006	0.0001	0.386
14	0.037	0.035	0.037	0.035	0.039	0.039	0.032	0.039	0.034	0.037	0.029	0.031	0.025	0.028	0.028	0.035	0.035	0.028	0.034	0.0010	0.011
33	0.147	0.118	0.135	0.102	0.158	0.127	0.136	0.148	0.166	0.138	0.126	0.132	0.091	0.155	0.101	0.128	0.141	0.090	0.130	0.0051	0.000
52	0.233	0.188	0.230	0.181	0.247	0.207	0.208	0.227	0.248	0.211	0.205	0.204	0.146	0.238	0.155	0.197	0.219	0.144	0.205	0.0073	0.000
73	0.253	0.197	0.248	0.213	0.262	0.238	0.233	0.250	0.267	0.230	0.223	0.222	0.167	0.259	0.168	0.206	0.243	0.155	0.224	0.0078	0.000
102	0.275	0.219	0.270	0.237	0.282	0.256	0.249	0.280	0.294	0.254	0.244	0.241	0.189	0.285	0.190	0.234	0.270	0.178	0.247	0.0079	0.000
132	0.279	0.230	0.287	0.248	0.293	0.267	0.262	0.292	0.316	0.271	0.259	0.256	0.200	0.301	0.205	0.249	0.275	0.192	0.260	0.0080	0.000
170	0.296	0.254	0.301	0.264	0.303	0.283	0.279	0.315	0.330	0.287	0.276	0.271	0.221	0.314	0.225	0.263	0.298	0.209	0.277	0.0077	0.000
217	0.310	0.280	0.314	0.275	0.315	0.300	0.300	0.340	0.347	0.310	0.297	0.290	0.243	0.336	0.244	0.286	0.313	0.229	0.296	0.0075	0.000
285	0.336	0.317	0.335	0.300	0.333	0.333	0.331	0.371	0.374	0.346	0.328	0.318	0.276	0.370	0.286	0.315	0.340	0.257	0.326	0.0072	0.000
360	0.355	0.344	0.355	0.324	0.349	0.362	0.370	0.410	0.407	0.370	0.360	0.350	0.320	0.399	0.327	0.347	0.365	0.298	0.356	0.0067	0.000
458	0.395	0.407	0.381	0.357	0.367	0.402	0.407	0.440	0.441	0.414	0.398	0.393	0.366	0.439	0.378	0.392	0.405	0.351	0.396	0.0062	0.000

PAR	ETR1	ETR2	ETR3	ETR4	ETR5	ETR6	ETR1	ETR2	ETR3	ETR4	ETR5	ETR6	ETR1	ETR2	ETR3	ETR4	ETR5	ETR6	MEAN	SEM	p Value *[rpn8a vs. WT]*
0	0.00	0.00	0.00	0.00	0.00	0.00	0.00	0.00	0.00	0.00	0.00	0.00	0.00	0.00	0.00	0.00	0.00	0.00	0.00	0.0000	#DIV/0!
14	2.80	3.00	2.80	2.90	2.70	2.80	4.00	3.80	3.60	3.60	3.70	3.50	4.00	3.60	3.70	3.50	3.50	3.70	3.40	0.1006	0.149
33	6.00	6.20	6.00	6.20	5.70	5.90	7.90	7.60	7.10	7.40	7.70	7.30	7.90	7.20	7.50	7.30	7.00	7.50	6.97	0.1714	0.068
52	8.70	9.10	8.60	9.20	8.10	8.60	11.70	11.10	10.30	10.90	11.20	10.90	12.00	10.60	11.30	10.90	10.50	11.40	10.28	0.2808	0.066
73	11.60	12.20	11.50	12.00	10.80	11.30	15.40	14.90	13.90	14.70	15.10	14.70	16.10	14.30	15.30	14.70	14.10	15.40	13.78	0.3909	0.059
102	15.70	16.90	15.80	16.40	14.60	15.60	21.40	20.30	18.70	20.50	21.00	20.40	22.30	19.60	21.60	20.20	19.70	21.60	19.02	0.5711	0.043
132	20.50	21.90	19.60	20.60	18.20	19.80	26.90	25.90	23.30	26.00	26.70	26.00	28.90	24.90	27.70	25.80	25.60	27.70	24.22	0.7531	0.047
170	25.50	27.40	24.60	25.60	22.50	24.50	33.60	32.30	29.50	32.80	33.60	33.00	36.30	31.90	34.90	32.80	32.00	35.50	30.46	0.9874	0.052
217	32.10	34.00	30.70	32.10	27.20	30.50	41.40	39.40	36.80	40.50	41.60	41.00	45.80	39.50	43.90	40.30	40.10	44.60	37.86	1.2494	0.053
285	39.40	41.40	37.50	38.80	32.50	35.90	50.20	48.60	45.10	48.40	50.80	50.30	56.40	47.90	52.50	49.40	49.70	55.50	46.13	1.5858	0.137
360	46.50	49.90	43.70	44.20	37.30	40.90	56.50	54.60	51.20	57.20	58.90	58.10	64.20	55.70	60.70	57.10	58.30	64.00	53.28	1.8102	0.104
458	50.80	52.90	49.80	48.40	41.90	44.30	63.70	63.30	57.50	63.70	66.60	64.60	72.40	62.70	67.80	62.70	65.20	70.90	59.40	2.0976	0.184

Fig.03

PAM data – *ppi2* (Biological replicate 1-3, n=7 each)

PAR	Y(II)1	Y(II)2	Y(II)3	Y(II)4	Y(II)5	Y(II)6	Y(II)7	Y(II)1	Y(II)2	Y(II)3	Y(II)4	Y(II)5	Y(II)6	Y(II)7	Y(II)1	Y(II)2	Y(II)3	Y(II)4	Y(II)5	Y(II)6	Y(II)7	MEAN	SEM
0	0.456	0.488	0.508	0.470	0.488	0.504	0.536	0.483	0.497	0.418	0.413	0.405	0.393	0.410	0.494	0.521	0.474	0.466	0.493	0.468	0.465	0.469	0.0085
14	0.330	0.382	0.356	0.349	0.425	0.356	0.353	0.417	0.437	0.369	0.363	0.329	0.317	0.320	0.420	0.437	0.419	0.388	0.413	0.292	0.340	0.372	0.0093
33	0.294	0.344	0.305	0.298	0.385	0.316	0.294	0.380	0.412	0.347	0.359	0.320	0.308	0.291	0.385	0.385	0.389	0.377	0.369	0.257	0.311	0.339	0.0092
52	0.264	0.322	0.277	0.274	0.360	0.288	0.268	0.375	0.405	0.329	0.335	0.312	0.289	0.293	0.350	0.360	0.352	0.341	0.353	0.235	0.290	0.318	0.0093
73	0.243	0.301	0.242	0.249	0.331	0.265	0.249	0.358	0.375	0.335	0.336	0.296	0.292	0.263	0.319	0.347	0.309	0.314	0.331	0.217	0.277	0.298	0.0093
102	0.212	0.274	0.203	0.214	0.290	0.223	0.206	0.329	0.351	0.312	0.301	0.276	0.242	0.243	0.270	0.312	0.282	0.284	0.285	0.189	0.255	0.264	0.0096
132	0.184	0.243	0.176	0.182	0.275	0.194	0.182	0.293	0.327	0.286	0.297	0.246	0.213	0.227	0.244	0.286	0.266	0.260	0.254	0.179	0.239	0.241	0.0097
170	0.162	0.215	0.147	0.151	0.229	0.172	0.159	0.266	0.298	0.251	0.241	0.228	0.199	0.202	0.228	0.260	0.244	0.216	0.235	0.148	0.200	0.212	0.0091
217	0.143	0.191	0.133	0.132	0.198	0.145	0.135	0.238	0.281	0.222	0.201	0.197	0.153	0.168	0.187	0.218	0.216	0.182	0.204	0.128	0.182	0.184	0.0086
285	0.105	0.161	0.105	0.107	0.156	0.118	0.119	0.205	0.230	0.185	0.174	0.175	0.121	0.149	0.153	0.182	0.182	0.163	0.169	0.110	0.141	0.153	0.0075
360	0.085	0.128	0.094	0.090	0.133	0.104	0.102	0.169	0.201	0.159	0.151	0.137	0.111	0.122	0.122	0.146	0.149	0.122	0.148	0.095	0.137	0.129	0.0062
458	0.062	0.097	0.065	0.077	0.107	0.084	0.089	0.135	0.166		0.127	0.112	0.081	0.095	0.103	0.123	0.141	0.113	0.126	0.082	0.105	0.105	0.0058

PAR	Y(NPQ)1	Y(NPQ)2	Y(NPQ)3	Y(NPQ)4	Y(NPQ)5	Y(NPQ)6	Y(NPQ)7	Y(NPQ)1	Y(NPQ)2	Y(NPQ)3	Y(NPQ)4	Y(NPQ)5	Y(NPQ)6	Y(NPQ)7	Y(NPQ)1	Y(NPQ)2	Y(NPQ)3	Y(NPQ)4	Y(NPQ)5	Y(NPQ)6	Y(NPQ)7	MEAN	SEM
0	0.007	0.008	0.008	0.007	0.008	0.008	0.007	0.007	0.007	0.007	0.008	0.008	0.007	0.007	0.008	0.008	0.008	0.000	0.008	0.000	0.000	0.006	0.0006
14	0.007	0.005	0.010	0.007	0.002	0.011	0.029	0.006	0.007	0.003	0.010	0.021	0.008	0.006	0.000	0.012	0.008	0.012	0.008	0.016	0.012	0.010	0.0014
33	0.041	0.030	0.068	0.051	0.010	0.051	0.061	0.028	0.019	0.004	0.008	0.008	0.008	0.041	0.016	0.016	0.020	0.012	0.016	0.027	0.016	0.026	0.0040
52	0.073	0.050	0.106	0.080	0.012	0.079	0.099	0.029	0.026	0.007	0.039	0.031	0.052	0.041	0.024	0.027	0.039	0.016	0.024	0.039	0.024	0.044	0.0060
73	0.093	0.077	0.145	0.111	0.057	0.100	0.143	0.029	0.036	0.005	0.039	0.033	0.010	0.054	0.035	0.035	0.055	0.016	0.035	0.051	0.031	0.057	0.0086
102	0.115	0.091	0.190	0.143	0.070	0.132	0.189	0.040	0.048	0.005	0.060	0.034	0.054	0.064	0.047	0.047	0.063	0.024	0.055	0.063	0.043	0.075	0.0108
132	0.129	0.112	0.218	0.172	0.072	0.144	0.223	0.043	0.049	0.007	0.052	0.044	0.057	0.065	0.051	0.063	0.059	0.024	0.071	0.075	0.047	0.085	0.0127
170	0.142	0.125	0.240	0.196	0.086	0.158	0.244	0.053	0.059	0.034	0.083	0.046	0.068	0.070	0.051	0.071	0.059	0.027	0.086	0.086	0.055	0.097	0.0136
217	0.145	0.129	0.252	0.214	0.089	0.163	0.264	0.056	0.053	0.036	0.088	0.059	0.084	0.092	0.055	0.086	0.067	0.027	0.094	0.094	0.063	0.105	0.0143
285	0.161	0.141	0.267	0.229	0.094	0.177	0.275	0.068	0.077	0.059	0.121	0.062	0.099	0.095	0.059	0.094	0.075	0.027	0.106	0.106	0.075	0.117	0.0146
360	0.174	0.156	0.276	0.234	0.107	0.189	0.288	0.073	0.088	0.061	0.106	0.075	0.109	0.108	0.063	0.102	0.078	0.031	0.114	0.114	0.075	0.125	0.0150
458	0.139	0.172	0.293	0.245	0.120	0.202	0.292	0.086	0.094		0.119	0.088	0.124	0.133	0.063	0.110	0.078	0.031	0.125	0.122	0.082	0.136	0.0156

PAR	ETR1	ETR2	ETR3	ETR4	ETR5	ETR6	ETR7	ETR1	ETR2	ETR3	ETR4	ETR5	ETR6	ETR7	ETR1	ETR2	ETR3	ETR4	ETR5	ETR6	ETR7	MEAN	SEM
0	0.00	0.00	0.00	0.00	0.00	0.00	0.00	0.00	0.00	0.00	0.00	0.00	0.00	0.00	0.00	0.00	0.00	0.00	0.00	0.00	0.00	0.00	0.0000
14	1.90	2.10	2.50	2.40	2.50	2.50	2.50	2.90	3.10	2.60	2.50	2.30	2.20	2.20	2.90	2.90	2.90	2.70	2.90	2.00	24.00	3.55	1.0005
33	3.40	4.50	4.60	4.50	4.40	4.70	4.40	6.30	6.80	5.70	5.90	5.30	5.10	4.80	6.40	6.40	6.40	6.20	6.10	4.20	5.10	5.30	0.2006
52	4.60	6.40	6.50	6.40	6.30	6.80	6.30	9.80	10.50	8.60	8.70	8.10	7.50	7.60	9.10	9.30	9.10	8.90	9.20	6.10	7.50	7.78	0.3232
73	5.80	7.90	7.70	8.00	8.00	8.50	8.00	13.10	13.70	12.20	12.30	10.80	10.60	9.60	11.60	12.70	11.30	11.50	12.10	7.90	10.10	10.16	0.4720
102	8.00	9.80	9.20	9.60	9.30	10.00	9.30	16.80	17.90	15.90	15.30	14.10	12.30	12.40	13.80	15.90	14.40	14.50	14.50	9.70	13.00	12.65	0.6312
132	9.00		10.20	10.60	10.50	11.30	10.50	19.30	21.60	18.90	19.60	16.30	14.10	15.00	16.10	18.90	17.50	17.20	16.70	11.80	15.80	15.05	0.8288
170	9.80	11.20	11.00	11.30	11.90	12.90	11.90	22.60	25.30	21.30	20.50	19.40	16.90	17.10	19.40	22.10	20.70	18.40	20.00	12.60	17.00	16.82	0.9928
217	12.70	11.60	12.90	12.80	13.10	14.10	13.10	25.90	30.50	24.10	21.80	21.40	16.60	18.20	20.30	23.60	23.50	19.70	22.20	13.90	19.70	18.65	1.1343
285	11.70	13.50	13.50	13.60	15.20	15.10	15.20	29.20	32.80	26.40	24.80	24.90	17.20	21.30	21.80	26.00	25.90	23.20	24.10	15.70	20.10	20.53	1.2814
360	11.60	16.80	15.20	14.50	16.50	16.80	16.50	30.40	36.20	28.60	27.20	24.70	20.00	22.00	22.00	26.30	26.80	22.00	26.70	17.00	24.60	22.02	1.3254
458		15.70	13.30	15.70	18.20	17.20	18.20	30.90	37.90		29.00	25.70	18.50	21.80	23.60	28.30	32.20	25.90	28.90	18.80	23.90	23.35	1.4907

Fig.03

PAM data – *rpn8axppi2* (Biological replicate 1-3, n=7 each)

PAR	Y(II)1	Y(II)2	Y(II)3	Y(II)4	Y(II)5	Y(II)6	Y(II)7	Y(II)1	Y(II)2	Y(II)3	Y(II)4	Y(II)5	Y(II)6	Y(II)7	Y(II)1	Y(II)2	Y(II)3	Y(II)4	Y(II)5	Y(II)6	Y(II)7	MEAN	SEM	p Value [*ppi2* vs. *rpn8axppi2*]
**0**	0.466	0.622	0.684	0.635	0.670	0.594	0.525	0.641	0.447	0.542	0.462	0.466	0.404	0.571	0.631	0.517	0.589	0.432	0.483	0.606	0.651	0.554	0.0185	0.000
**14**	0.411	0.441	0.448	0.473	0.506	0.428	0.423	0.469	0.388	0.446	0.390	0.448	0.354	0.423	0.478	0.440	0.439	0.352	0.436	0.380	0.448	0.430	0.0084	0.000
**33**	0.373	0.420	0.412	0.471	0.513	0.408	0.411	0.466	0.364	0.435	0.380	0.391	0.372	0.418	0.455	0.430	0.414	0.335	0.417	0.344	0.418	0.412	0.0092	0.000
**52**	0.337	0.387	0.414	0.454	0.500	0.368	0.384	0.462	0.326	0.428	0.362	0.340	0.342	0.407	0.417	0.402	0.405	0.322	0.396	0.352	0.433	0.392	0.0102	0.000
**73**	0.293	0.336	0.382	0.414	0.457	0.323	0.339	0.425	0.294	0.400	0.344	0.352	0.340	0.374	0.385	0.367	0.372	0.303	0.361	0.360	0.441	0.365	0.0096	0.000
**102**	0.261	0.303	0.327	0.358	0.406	0.272	0.286	0.386	0.253	0.358	0.311	0.322	0.289	0.337	0.358	0.336	0.344	0.269	0.343	0.342	0.421	0.328	0.0098	0.000
**132**	0.227	0.285	0.317	0.329	0.377	0.252	0.262	0.364	0.204	0.324	0.288	0.261	0.230	0.312	0.333	0.320	0.333	0.257	0.318	0.317	0.405	0.301	0.0110	0.000
**170**	0.200	0.252	0.293	0.302	0.343	0.228	0.245	0.331	0.202	0.289	0.250	0.291	0.260	0.286	0.302	0.289	0.306	0.234	0.290	0.299	0.389	0.280	0.0097	0.000
**217**	0.175	0.219	0.268	0.250	0.295	0.213	0.213	0.284	0.177	0.244	0.208	0.191	0.232	0.250	0.263	0.250	0.261	0.196	0.249	0.263	0.355	0.241	0.0091	0.000
**285**	0.146	0.176	0.235	0.213	0.232	0.162	0.163	0.232	0.127	0.221	0.184	0.229	0.169	0.195	0.213	0.218	0.216	0.163	0.204	0.230	0.308	0.202	0.0085	0.000
**360**	0.095	0.146	0.195	0.174	0.185	0.134	0.136	0.183	0.093	0.176	0.141	0.173	0.119	0.169	0.169	0.164	0.171	0.117	0.164	0.181	0.261	0.159	0.0080	0.005
**458**	0.115	0.111	0.160	0.145	0.164	0.105	0.110	0.155	0.104	0.148	0.113	0.174	0.142	0.136	0.144	0.134	0.141	0.108	0.133			0.134	0.0048	0.001

PAR	Y(NPQ)1	Y(NPQ)2	Y(NPQ)3	Y(NPQ)4	Y(NPQ)5	Y(NPQ)6	Y(NPQ)7	Y(NPQ)1	Y(NPQ)2	Y(NPQ)3	Y(NPQ)4	Y(NPQ)5	Y(NPQ)6	Y(NPQ)7	Y(NPQ)1	Y(NPQ)2	Y(NPQ)3	Y(NPQ)4	Y(NPQ)5	Y(NPQ)6	Y(NPQ)7	MEAN	SEM	p Value [*ppi2* vs. *rpn8axppi2*]
**0**	0.008	0.006	0.006	0.006	0.007	0.007	0.008	0.011	0.008	0.013	0.008	0.008	0.007	0.013	0.008	0.008	0.008	0.000	0.008	0.008	0.008	0.008	0.0006	0.116
**14**	0.005	0.032	0.032	0.032	0.027	0.028	0.032	0.030	0.027	0.024	0.006	0.004	0.030	0.033	0.020	0.012	0.012	0.012	0.008	0.016	0.016	0.021	0.0022	0.000
**33**	0.010	0.079	0.064	0.054	0.053	0.059	0.044	0.048	0.038	0.034	0.035	0.037	0.028	0.049	0.047	0.020	0.024	0.020	0.008	0.024	0.027	0.038	0.0039	0.043
**52**	0.019	0.146	0.106	0.093	0.095	0.101	0.067	0.065	0.059	0.049	0.038	0.065	0.007	0.060	0.094	0.039	0.043	0.031	0.020	0.031	0.039	0.060	0.0074	0.096
**73**	0.078	0.208	0.168	0.141	0.135	0.143	0.097	0.090	0.079	0.060	0.057	0.055	0.030	0.078	0.129	0.055	0.067	0.039	0.027	0.043	0.059	0.088	0.0104	0.031
**102**	0.109	0.243	0.221	0.187	0.167	0.174	0.128	0.105	0.101	0.088	0.077	0.050	0.031	0.098	0.153	0.067	0.078	0.047	0.027	0.059	0.075	0.109	0.0130	0.058
**132**	0.123	0.263	0.229	0.205	0.186	0.185	0.140	0.122	0.135	0.106	0.088	0.091	0.085	0.124	0.157	0.067	0.086	0.051	0.035	0.075	0.086	0.126	0.0128	0.032
**170**	0.127	0.292	0.246	0.223	0.206	0.199	0.145	0.147	0.143	0.142	0.110	0.070	0.065	0.144	0.173	0.071	0.094	0.055	0.039	0.082	0.094	0.137	0.0144	0.059
**217**	0.132	0.315	0.265	0.266	0.249	0.209	0.158	0.183	0.157	0.160	0.143	0.118	0.076	0.172	0.192	0.078	0.106	0.059	0.047	0.098	0.110	0.157	0.0156	0.022
**285**	0.153	0.349	0.295	0.288	0.287	0.243	0.191	0.211	0.192	0.185	0.156	0.076	0.103	0.213	0.216	0.086	0.122	0.071	0.059	0.118	0.129	0.178	0.0175	0.013
**360**	0.105	0.371	0.324	0.313	0.319	0.264	0.198	0.243	0.208	0.211	0.189	0.102	0.139	0.234	0.231	0.098	0.137	0.075	0.067	0.141	0.153	0.196	0.0188	0.006
**458**	0.177	0.395	0.357	0.330	0.332	0.286	0.212	0.264	0.198	0.226	0.212	0.102	0.107	0.257	0.247	0.106	0.145	0.075	0.075			0.216	0.0218	0.006

PAR	ETR1	ETR2	ETR3	ETR4	ETR5	ETR6	ETR7	ETR1	ETR2	ETR3	ETR4	ETR5	ETR6	ETR7	ETR1	ETR2	ETR3	ETR4	ETR5	ETR6	ETR7	MEAN	SEM	p Value [*ppi2* vs. *rpn8axppi2*]
**0**	0.00	0.000	0.000	0.000	0.000	0.000	0.00	0.00	0.000	0.000	0.000	0.000	0.000	0.00	0.00	0.00	0.00	0.00	0.00	0.00	0.00	0.00	0.0000	#DIV/0!
**14**	2.90	3.10	3.10	3.30	3.50	3.00	3.00	3.30	2.70	3.10	2.70	3.10	2.50	3.00	3.30	3.10	3.10	2.50	3.10	2.70	3.10	3.01	0.0559	0.603
**33**	5.60	6.30	6.20	7.10	7.70	6.10	6.20	7.70	6.00	7.20	6.30	6.50	6.10	6.90	7.50	7.10	6.80	5.50	6.90	5.70	6.90	6.59	0.1411	0.000
**52**	7.90	9.10	9.70	10.70	11.80	8.70	9.00	12.00	8.50	11.10	9.40	8.80	8.90	10.60	10.80	10.50	10.50	8.40	10.30	9.20	11.30	9.87	0.2534	0.000
**73**	9.40	10.70	12.20	13.30	14.60	10.30	10.90	15.50	10.70	14.60	12.60	12.80	12.40	13.70	14.10	13.40	13.60	11.00	1.20	13.10	16.10	12.20	0.6556	0.018
**102**	11.80	13.60	14.70	16.10	18.30	12.30	12.90	19.70	12.90	18.20	15.90	16.40	14.70	17.20	18.30	17.10	17.50	13.70	17.50	17.50	21.50	16.09	0.5507	0.000
**132**	13.20	16.50	18.40	19.10	21.90	14.60	15.20	24.10	13.50	21.40	19.00	17.20	15.20	20.60	22.00	21.10	22.00	16.90	21.00	20.90	26.70	19.07	0.7654	0.001
**170**	15.00	18.90	22.00	22.70	25.80	17.10	18.40	28.20	17.10	24.60	21.30	24.70	22.10	24.30	25.70	24.50	26.00	19.90	24.60	25.40	33.10	22.92	0.8994	0.000
**217**	17.00	21.30	25.90	24.20	28.70	20.70	20.70	30.80	19.20	26.50	22.50	20.80	25.20	27.20	28.60	27.10	28.30	21.20	27.00	28.50	38.50	25.23	1.0303	0.000
**285**	18.60	22.60	30.10	27.30	29.80	20.70	20.90	32.90	18.10	31.40	26.30	32.60	24.10	27.80	30.40	31.10	30.80	23.20	29.00	32.80	43.80	27.82	1.2791	0.000
**360**	15.40	23.70	31.40	28.10	29.90	21.60	22.00	35.40	16.70	31.70	25.40	31.10	21.40	30.50	30.50	29.50	30.70	21.10	29.60	32.60	47.00	27.87	1.4903	0.007
**458**	23.50	22.60	32.60	29.60	33.60	21.40	22.40	35.40	23.70	33.90	25.80	39.90	32.40	31.20	32.90	30.60	32.30	24.80	30.30			29.42	1.1653	0.004

Fig.04

Uncropped Western Blots (same membrane, serial antibody detection) – Rep 1

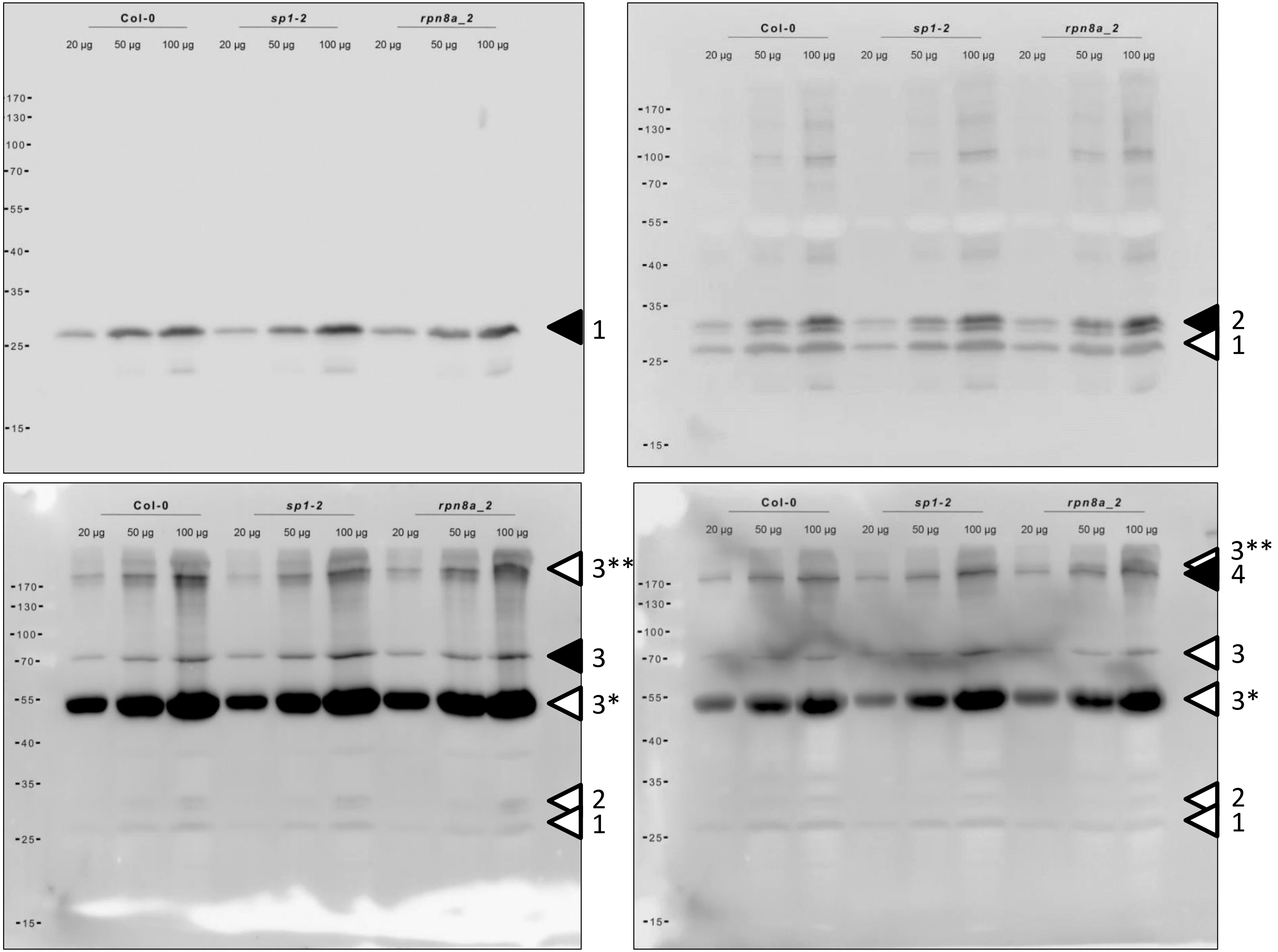

1 – Lhcb4.1
2 – OEC33 (double band)
3 – Toc75
3* – cross reaction with RBCL
3** – cross reaction (Toc159?)
4 – Toc132

Fig.04

Uncropped Western Blots (same membrane, serial antibody detection) – Rep 1

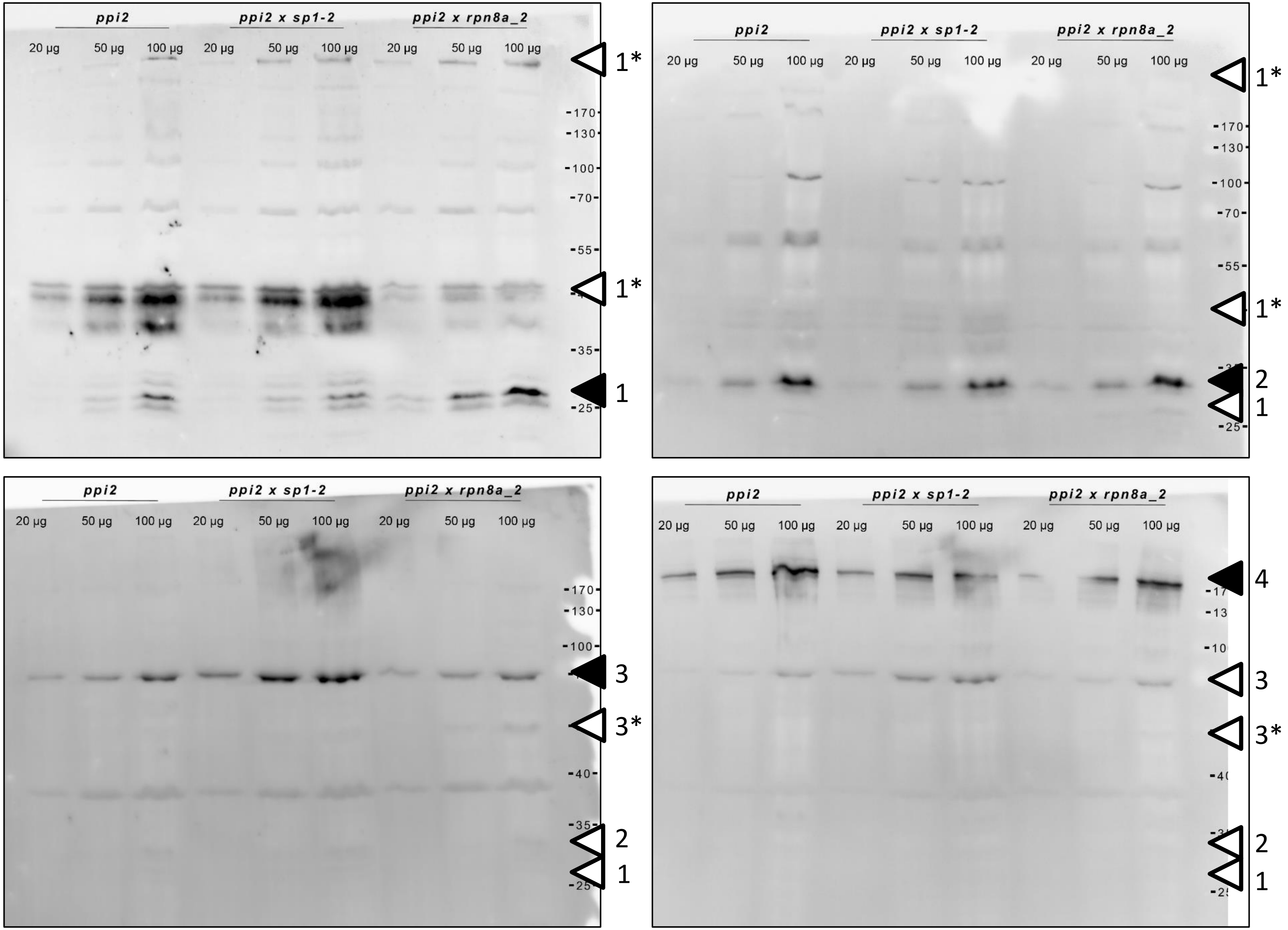

1 – Lhcb4.1
1* – unknown cross reaction
2 – OEC33 (double band)
3 – Toc75
3* – cross reaction with RBCL
4 – Toc132

Fig.04

Uncropped Western Blots (same membrane, serial antibody detection) – Rep 2

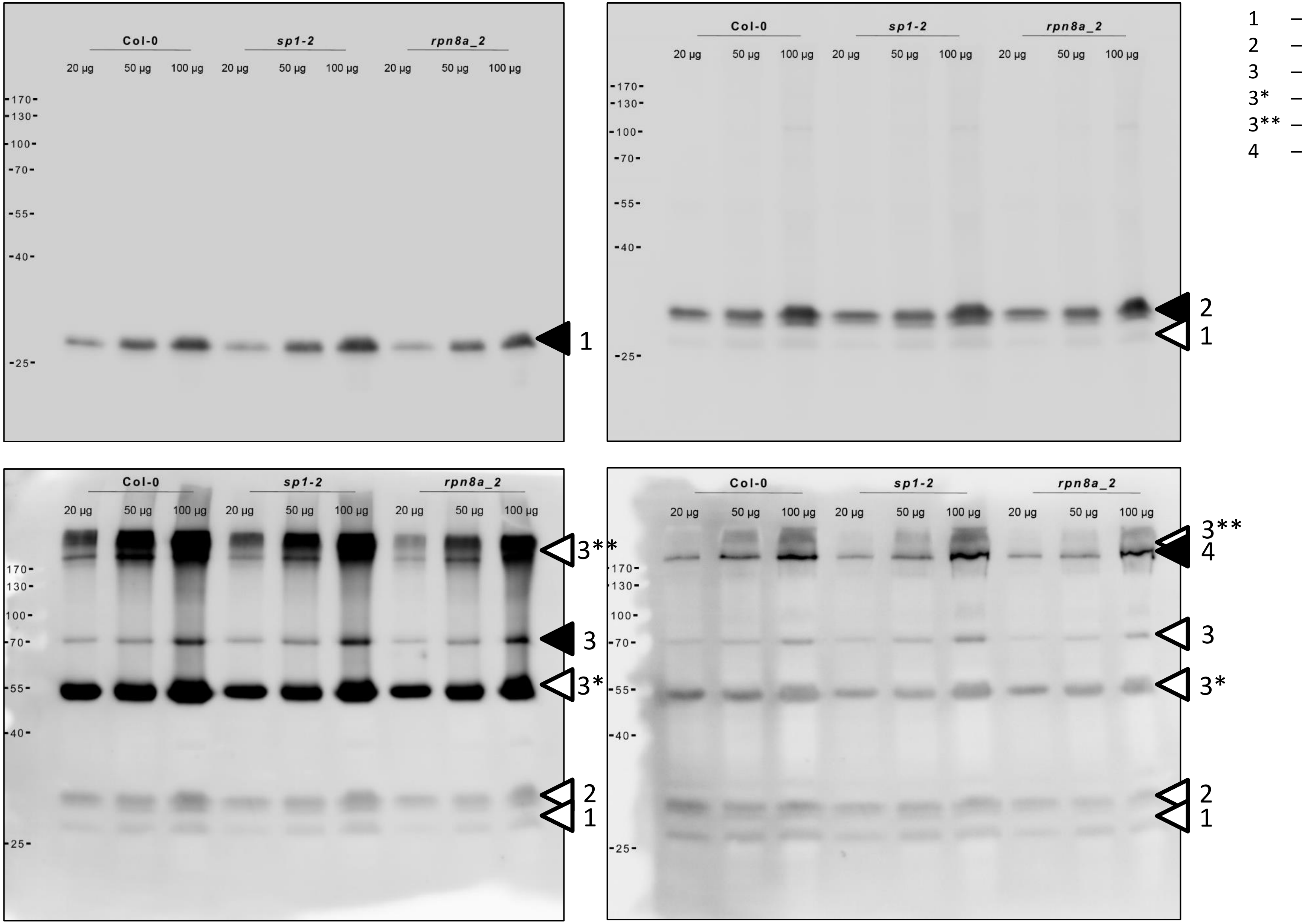

1 – Lhcb4.1
2 – OEC33 (double band)
3 – Toc75
3* – cross reaction with RBCL
3** – cross reaction (Toc159?)
4 – Toc132

Fig.04

Uncropped Western Blots (same membrane, serial antibody detection) – Rep 2

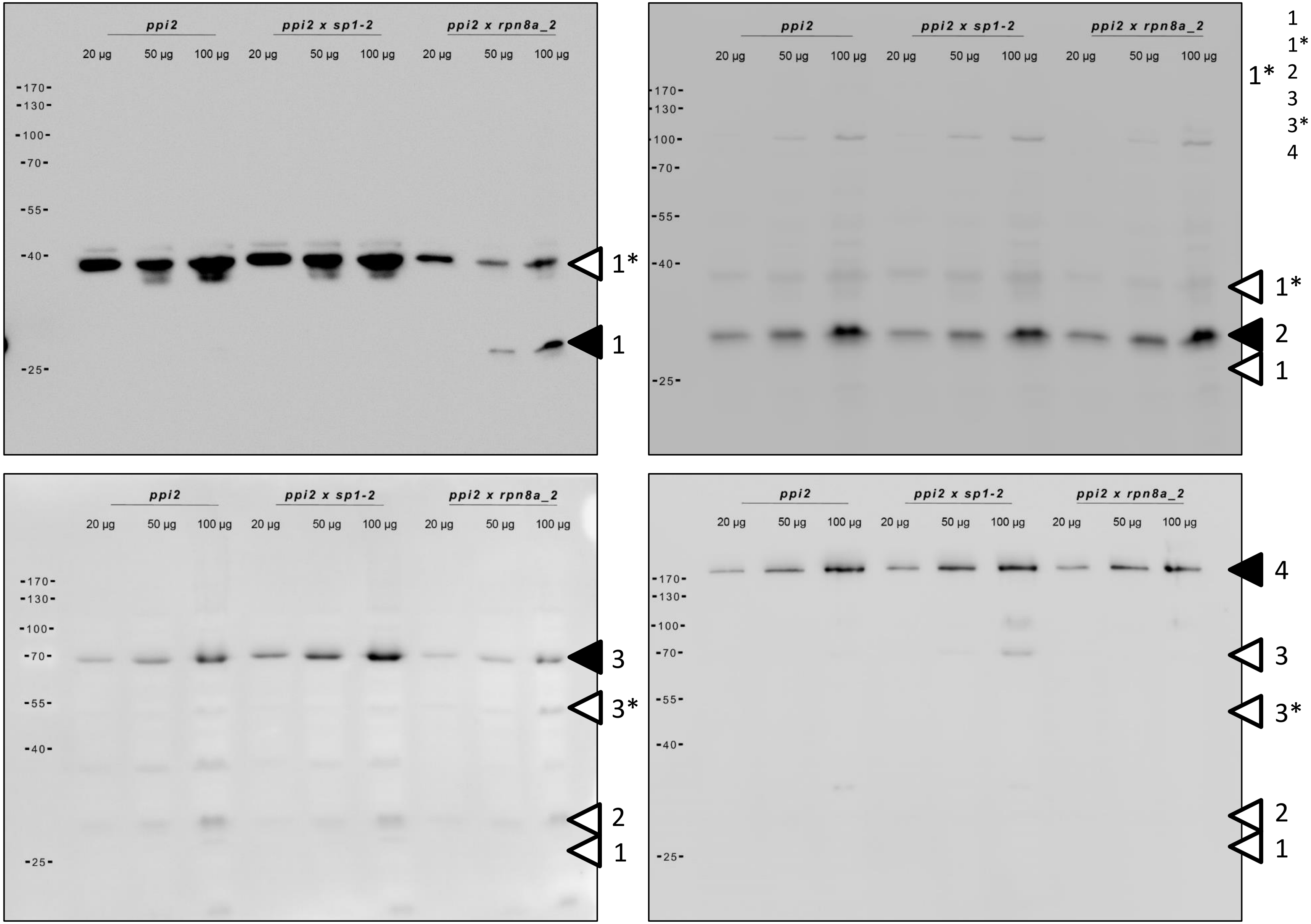

1 – Lhcb4.1
1* – unknown cross reaction
2 – OEC33 (double band)
3 – Toc75
3* – cross reaction with RBCL
4 – Toc132

Fig.04

Uncropped Western Blots (same membrane, serial antibody detection) – Rep 3

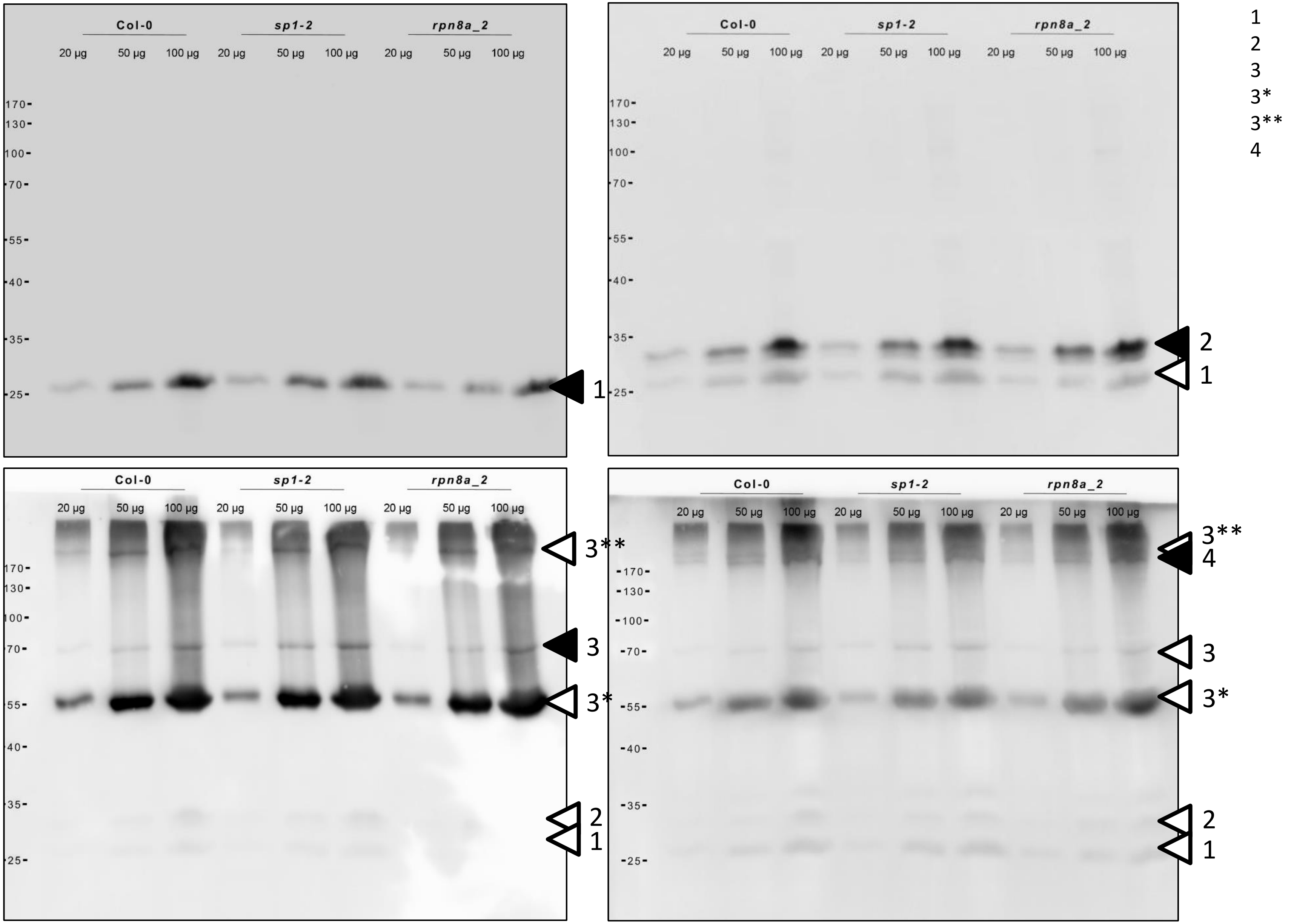

1 – Lhcb4.1
2 – OEC33 (double band)
3 – Toc75
3* – cross reaction with RBCL
3** – cross reaction (Toc159?)
4 – Toc132

Fig.04

Uncropped Western Blots (same membrane, serial antibody detection) – Rep 3

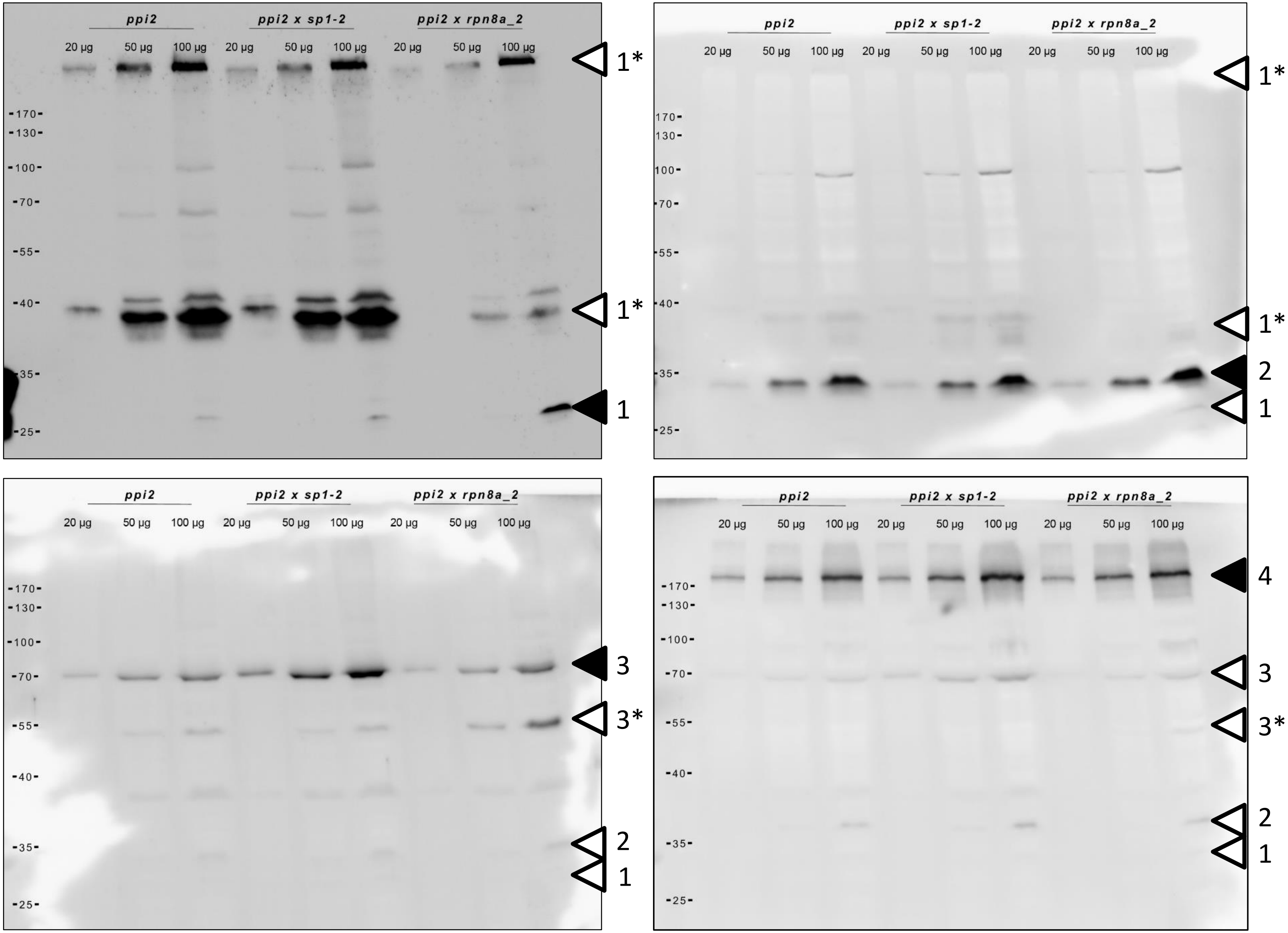

1 – Lhcb4.1
1* – unknown cross reaction
2 – OEC33 (double band)
3 – Toc75
3* – cross reaction with RBCL
4 – Toc132

Fig.05

Real-time PCR data of photosynthetic genes (3 Biological replicates, each measured in 3 technical replicates)

gene / line	ΔCt Rep 1	ΔCt Rep 2	ΔCt Rep 3	ΔCt Rep 4	p value [**contr.** vs mut]
psaJ	*ATCG00630*				
**WT**	**-6.20**	**-7.17**	**-6.83**	**-6.67**	
rpn8a	-7.10	-7.80	-7.73	-6.57	0.0878
ppi2	**-3.97**	**-5.03**	**-4.17**	**-3.70**	
rpn 8ax ppi2	-4.40	-5.47	-5.23	-3.33	0.2342
psbA	*ATCG00020*				
**WT**	**-8.20**	**-9.43**	**-8.67**	**-10.03**	
rpn8a	-8.97	-10.03	-9.13	-9.33	0.2794
ppi2	**-4.95**	**-5.60**	**-5.37**	**-6.40**	
rpn 8ax ppi2	-5.67	-6.22	-4.90	-6.17	0.5400
psbN	*ATCG00700*				
**WT**	**-5.03**	**-5.63**	**-6.47**	**-6.01**	
rpn8a	-6.17	-6.53	-6.57	-6.10	0.1774
ppi2	**-2.93**	**-3.57**	**-3.03**	**-2.60**	
rpn 8ax ppi2	-2.63	-3.57	-2.30	-2.27	0.4668
ndhF	*ATCG01110*				
**WT**	**-1.98**	**-2.83**	**-2.73**		
rpn8a	-2.80	-2.87	-2.75		0.3438
ppi2	**-3.27**	**-4.23**	**-3.77**		
rpn 8ax ppi2	-2.83	-3.83	-3.37		0.3640
RbcL	*ATCG00490*				
**WT**	**-6.10**	**-6.10**	**-5.87**		
rpn8a	-6.90	-6.67	-6.30		0.0349
ppi2	**-2.92**	**-3.91**	**-3.27**		
rpn 8ax ppi2	-2.37	-3.20	-2.70		0.1831

gene / line	ΔCt Rep 1	ΔCt Rep 2	ΔCt Rep 3	ΔCt Rep 4	p value [**contr.** vs mut]
RbcS	*AT1G67090*				
**WT**	**-4.73**	**-6.47**	**-6.20**		
rpn8a	-5.03	-6.40	-6.77		0.7426
ppi2	**-2.80**	**-4.40**	**-3.93**		
rpn 8ax ppi2	-2.27	-3.80	-3.37		0.4382
Lhcb4	*AT5G01530*				
**WT**	**-1.80**	**-2.08**	**-1.80**		
rpn8a	-1.90	-2.20	-2.20		0.2094
ppi2	**0.65**	**0.27**	**0.63**		
rpn 8ax ppi2	0.70	0.13	0.67		0.9443
Lhcb5	*AT4G10340*				
**WT**	**-2.23**	**-2.33**	**-2.45**		
rpn8a	-2.30	-2.57	-2.60		0.2576
ppi2	**0.25**	**-0.37**	**-0.13**		
rpn 8ax ppi2	0.28	-0.20	0.07		0.5896
ZDS	*AT3G04870*				
**WT**	**2.60**	**1.47**	**2.07**		
rpn8a	2.37	2.10	1.77		0.9339
ppi2	**2.53**	**1.33**	**1.93**		
rpn 8ax ppi2	3.07	1.93	1.92		0.5099

Fig.06

Proteasome activity assay (AMC assay) of three biological replicates and three technical replicates,
Excitation 360 nm, Emission 440 nm, 0 - 121 min

time [min]	0.00	4.31	8.59	12.87	17.16	21.45	25.77	30.08	34.41	38.81	43.19	47.59	52.01	56.44
ppi2 1-0µM-1	12719	12253	11700	11660	11628	12123	12320	12688	13268	13683	14490	14853	15866	16714
ppi2 1-0µM-2	13101	12281	11850	11849	11802	12021	12355	12932	13366	14072	14351	15380	16013	16808
ppi2 1-0µM-3	13035	12348	11938	11845	11837	12345	12501	12660	13013	13402	13845	14743	15234	16084
ppi2 2-0µM-1	12745	11966	11532	11561	11480	11862	12278	12301	12831	13145	13810	14322	14951	15872
ppi2 2-0µM-2	13382	12460	12022	12175	11771	12245	12409	12821	13297	13680	14108	14749	15299	15989
ppi2 2-0µM-3	13627	13151	12899	12722	12939	13106	13331	13862	14325	14909	15385	16057	16812	17776
ppi2 3-0µM-1	14353	13582	13325	13123	12848	13069	13428	13850	14157	14387	15062	15682	16260	16977
ppi2 3-0µM-2	14361	13512	12668	12536	12458	12376	12787	12919	13168	13848	14214	14959	15757	16583
ppi2 3-0µM-3	14963	13368	13077	12730	12697	12656	12991	13021	13234	13640	14166	14672	15068	15628
blank1A	11175	10678	10404	10407	10329	10107	10190	10047	10030	10020	10349	10259	10419	10452
ppi2 1-0.5µM-1	12645	12097	11610	11567	11281	11390	11463	11441	11717	12023	12212	12531	13114	13450
ppi2 1-0.5µM-2	12605	12231	11947	11923	11820	12036	12264	12571	12609	12888	13357	13675	13920	14637
ppi2 1-0.5µM-3	12114	11670	11042	10786	10672	10686	10867	10934	11190	11125	11808	11871	12108	12832
ppi2 2-0.5µM-1	13162	12357	12301	12201	11735	11846	11926	11885	12058	12223	12664	12648	12980	13279
ppi2 2-0.5µM-2	12843	12187	11749	11553	11429	11261	11435	11683	11714	11954	12197	12422	12723	13029
ppi2 2-0.5µM-3	13181	12397	12044	11836	11450	11543	11522	11634	11903	11989	12388	12694	12760	13264
ppi2 3-0.5µM-1	13936	13180	12536	12326	11854	11588	11638	11737	11617	11869	12074	12032	12210	12342
ppi2 3-0.5µM-2	14425	13397	12937	12675	12004	12142	12180	12102	12308	12276	12434	12820	12824	13031
ppi2 3-0.5µM-3	14557	13858	13061	12512	12492	12235	12239	11961	12341	12122	12374	12389	12667	13062
blank2A	10627	10221	10138	9874	9854	9899	10040	9862	9900	10046	10206	10300	10393	10523
ppi2 1-2µM-1	12359	11856	11528	11207	10992	10936	11013	10996	11138	11266	11301	11746	12040	12428
ppi2 1-2µM-2	12801	12099	11810	11454	11256	11325	11357	11173	11253	11561	11826	11955	12264	12537
ppi2 1-2µM-3	13048	12272	11916	11642	11221	11341	11458	11546	11466	11590	11970	11930	12366	12686
ppi2 2-2µM-1	12717	12121	11930	11722	11429	11699	11636	11681	11830	11982	12158	12375	12708	12952
ppi2 2-2µM-2	13111	12386	11991	11542	11141	11110	10970	11203	11172	11336	11527	11658	11816	12071
ppi2 2-2µM-3	12927	12309	12142	12036	11739	11752	11783	11860	11877	12217	12138	12581	12824	13022
ppi2 3-2µM-1	13723	13248	12642	12543	12431	12351	12021	12227	12361	12324	12575	12621	12797	12925
ppi2 3-2µM-2	13936	13034	12802	12220	11970	11890	11938	12001	11919	11991	11987	12021	12123	12544
ppi2 3-2µM-3	14175	13350	12842	12106	11788	11786	11577	11820	11647	11970	11768	12025	12202	12476
blank3A	10508	10185	10042	9952	9612	9822	9917	9627	9976	10017	10162	10305	10198	10531
ppi2 1-80µM-1	12150	11990	11558	11315	11166	11015	10970	11110	10826	11148	11323	11549	11566	11808
ppi2 1-80µM-2	12315	11845	11580	11406	11298	11262	11440	11434	11619	11841	11955	12120	12335	12667
ppi2 1-80µM-3	12081	11715	11280	11076	10846	10507	10507	10523	10495	10414	10489	10672	10800	10915
ppi2 2-80µM-1	12481	11968	11644	11482	11370	11203	11385	11296	11451	11405	11494	11591	11792	11974
ppi2 2-80µM-2	12267	11842	11623	11548	11239	11179	11211	11337	11571	11567	11779	12062	12170	12595
ppi2 2-80µM-3	12937	12500	12100	11733	11585	11596	11735	11700	11989	11823	11918	12149	12264	12540
ppi2 3-80µM-1	12876	12437	12151	11863	11755	11668	11589	11563	11481	11708	11559	11745	11932	11878
ppi2 3-80µM-2	13263	12565	12519	12089	11889	11890	11883	11763	11867	11834	11816	11831	11835	12071
ppi2 3-80µM-3	13974	13186	12710	12425	12113	11759	11707	11792	11800	11724	11646	11965	11911	11864
blank1B	838	770	796	799	811	863	877	884	893	898	899	913	897	926
xrpn8a 1-0µM-1	12662	12645	12785	12573	12935	13400	13919	14461	15348	15987	16577	17820	18518	19812
xrpn8a 1-0µM-2	12827	12933	12885	12641	12893	13278	13944	13979	14822	15473	16000	16726	17462	18446
xrpn8a 1-0µM-3	12950	13194	12998	13124	13162	13918	14359	14790	15495	16223	16646	17554	18399	19158
xrpn8a 2-0µM-1	12607	12111	11907	11800	11920	12071	12466	12732	13252	13675	14298	14906	15533	16266
xrpn8a 2-0µM-2	12762	12678	12686	12731	13051	13485	13791	14691	14912	16002	16229	17361	18169	19013
xrpn8a 2-0µM-3	12872	12708	12786	12748	12952	13623	13720	14232	14619	15248	16106	16843	17533	18617
xrpn8a 3-0µM-1	13373	13504	13239	13041	13017	13430	13838	14088	14455	14925	15514	16105	16962	17616
xrpn8a 3-0µM-2	12936	12863	12603	12513	12755	13111	13119	14038	14226	14829	15216	16255	16798	17645
xrpn8a 3-0µM-3	14311	13971	13767	13487	13470	13668	13769	14195	14652	14955	15562	16275	16725	17562
blank2B	1174	1100	1063	952	972	923	898	915	922	921	883	907	893	861
xrpn8a 1-0.5µM-1	12316	11765	11221	11252	10978	11173	11230	11357	11432	11809	12148	12375	12714	13060
xrpn8a 1-0.5µM-2	12744	12366	12351	12102	12259	12481	12659	12568	13054	13305	13736	13918	14246	14902
xrpn8a 1-0.5µM-3	12729	12242	12086	12048	11790	12154	12078	12114	12570	12884	13011	13310	13845	14194
xrpn8a 2-0.5µM-1	12337	12141	11633	11107	11192	11159	11159	11398	11398	11641	12005	12229	12531	12964
xrpn8a 2-0.5µM-2	12708	12269	12127	12108	11939	11983	12126	12536	12773	13246	13543	13886	14347	14889
xrpn8a 2-0.5µM-3	13052	12190	11746	11801	11661	11747	11553	11679	11857	12090	12177	12448	13120	13256
xrpn8a 3-0.5µM-1	13237	12948	12690	12418	12358	12603	12677	12852	12961	12977	13382	13817	13961	14243
xrpn8a 3-0.5µM-2	13809	13553	13247	13092	13047	13134	13545	13645	13848	13659	14182	14463	14940	15261
xrpn8a 3-0.5µM-3	13995	13539	13243	12991	12865	12763	12834	13148	13425	13492	13832	14042	14230	14720
blank3B	1980	1958	1933	1849	1839	1864	1836	1845	1821	1837	1866	1851	1893	1872
xrpn8a 1-2µM-1	12072	11478	11298	11141	10777	11276	11114	11393	11777	12151	12122	12643	13257	13652
xrpn8a 1-2µM-2	12083	11540	11330	11333	11195	11347	11357	11630	11760	12121	12578	12885	13396	13769
xrpn8a 1-2µM-3	12099	11884	11435	11144	11118	11062	11157	10906	11160	11314	11296	11749	11886	12165
xrpn8a 2-2µM-1	12740	12073	11664	11422	11080	11230	11274	11440	11267	11652	11769	12010	12400	12432
xrpn8a 2-2µM-2	12935	12354	12346	12293	12132	12302	12386	12625	12819	13279	13602	13844	14274	14553
xrpn8a 2-2µM-3	12031	11363	11236	11119	10964	11076	11105	11444	11591	11831	11845	12266	12713	12942
xrpn8a 3-2µM-1	13373	13105	12892	12731	12487	12736	12718	12779	12885	13041	13024	13381	13565	13870
xrpn8a 3-2µM-2	13609	12905	12678	12477	12186	12525	12743	12863	12942	12951	13060	13249	13465	13892
xrpn8a 3-2µM-3	14147	13471	13005	12532	12369	12202	12248	12203	12323	12450	12795	12712	13061	13411
blank	807	790	792	783	778	792	777	804	791	759	789	783	774	741
xrpn8a 1-80µM-1	12230	11273	10940	10786	10506	10382	10347	10430	10403	10548	10687	10844	11095	11446
xrpn8a 1-80µM-2	12008	11652	11360	10876	10667	10798	10542	10588	10851	10911	11055	11171	11237	11629
xrpn8a 1-80µM-3	11950	11249	10908	10514	10403	10505	10427	10229	10432	10532	10833	10794	10882	11361
xrpn8a 2-80µM-1	12324	12075	12184	11629	11827	11762	11719	12099	12249	12395	12523	12690	13144	13377
xrpn8a 2-80µM-2	12177	11336	11201	10768	10829	10568	10341	10566	10591	10404	10628	10837	10829	11150
xrpn8a 2-80µM-3	12097	11576	11405	11049	10866	10980	11096	11009	11297	11151	11433	11787	11769	11982
xrpn8a 3-80µM-1	13164	12263	11977	11749	11394	11518	11364	11400	11300	11539	11599	11515	11806	11951
xrpn8a 3-80µM-2	13320	12638	12061	11807	11627	11614	11635	11642	11429	11435	11611	11737	12039	12249
xrpn8a 3-80µM-3	13601	12977	12270	11921	11816	11767	11605	11427	11621	11649	11909	11960	11973	12335
leer	77	67	67	64	69	71	70	75	65	82	74	70	75	64

time [min]	60.87	65.28	69.69	74.10	78.46	82.82	87.16	91.50	95.86	100.21	104.55	108.90	113.27	117.61	121.95
ppi2 1-0µM-1	17569	18575	19644	21238	22404	23869	25914	28080	29901	32277	35416	37215	39475	42158	44470
ppi2 1-0µM-2	17906	18752	20013	21527	22704	24356	26539	28432	30174	32966	35688	37741	40641	42886	45125
ppi2 1-0µM-3	16952	17967	18768	19786	21103	22542	24293	26377	27866	30308	33256	34803	37626	40035	42206
ppi2 2-0µM-1	16752	17633	18561	19842	20790	22595	23659	25818	27784	29818	32427	33926	36594	38878	41505
ppi2 2-0µM-2	16949	17899	18670	19770	21213	22585	24139	25351	27402	29514	32083	34061	36278	38785	40849
ppi2 2-0µM-3	18605	19328	20406	21573	22482	23799	25351	27079	28955	31179	32968	35503	37113	39552	42261
ppi2 3-0µM-1	17865	18896	20348	21741	22985	25128	26212	28459	29805	31713	34414	35620	38026	39606	41123
ppi2 3-0µM-2	17503	18613	20062	21797	23058	25124	26565	28278	30686	32760	34511	36685	38483	40132	41836
ppi2 3-0µM-3	16610	17284	17870	18577	19695	20693	21958	23390	25357	26870	28890	30815	32952	34479	36245
blank1A	10418	10665	10812	10986	11184	11257	11259	11365	11418	11525	11688	11705	12007	12209	12465
ppi2 1-0.5µM-1	13895	14294	14847	15465	16306	17374	18251	19232	20774	22069	23941	25100	26776	28312	30043
ppi2 1-0.5µM-2	15083	15743	16515	17191	17992	18866	19895	20863	22509	23704	25347	26663	28056	29986	31558
ppi2 1-0.5µM-3	13181	14021	14301	15156	15929	17048	18251	19161	20668	22048	23559	25148	26818	28427	29772
ppi2 2-0.5µM-1	13676	13983	14496	14827	15466	15903	16669	17117	17979	18830	20295	21202	22519	23339	25130
ppi2 2-0.5µM-2	13350	13662	14100	14749	15302	16040	16635	17900	18468	19764	20952	22212	23262	24891	26761
ppi2 2-0.5µM-3	13728	14273	14368	15191	15791	16506	17293	17793	19054	20133	21171	22687	23880	25566	26764
ppi2 3-0.5µM-1	12512	12846	13151	13491	13875	14457	14847	15052	15919	16625	17401	18241	19232	19795	21183
ppi2 3-0.5µM-2	13483	13903	14299	14886	15694	16527	17301	18283	19139	20198	20980	21947	22898	23898	25060
ppi2 3-0.5µM-3	13098	13435	13845	14178	14427	14887	15633	16277	16655	17354	18495	19499	20202	21317	22470
blank2A	10654	10769	11051	11201	11104	11238	11478	11538	11570	11843	12256	12151	12393	12539	12770
ppi2 1-2µM-1	12637	13118	13707	14258	15022	15585	16219	16881	18023	19648	20646	21745	23196	24469	25636
ppi2 1-2µM-2	12990	13228	13565	14420	14738	15352	16071	16787	17854	18758	20023	20638	21973	23220	24425
ppi2 1-2µM-3	12892	13363	13926	14477	14923	15680	16246	16926	17844	18806	20056	21491	22803	23695	25072
ppi2 2-2µM-1	13211	13661	14089	14464	14884	15776	16137	16766	17463	18490	19009	20306	21473	22446	23465
ppi2 2-2µM-2	12427	12765	13094	13509	13848	14612	15289	15701	16328	17467	18157	19519	20246	21090	22255
ppi2 2-2µM-3	13446	13623	14253	14683	15118	15864	16573	16987	17544	18815	19691	20972	21692	22837	24031
ppi2 3-2µM-1	13050	13380	13395	13738	14089	14738	15051	15576	15950	16717	17228	18101	18802	19459	20431
ppi2 3-2µM-2	12692	13050	13169	13424	13853	14273	14651	15460	16168	16733	17351	18297	18869	19860	20823
ppi2 3-2µM-3	12426	12947	13039	13488	13778	14165	14671	15056	15482	16192	16933	17794	18526	19246	19923
blank3A	10685	10926	11055	11230	11777	11672	11929	11814	11916	12276	12173	12286	12334	12554	12896
ppi2 1-80µM-1	11948	12332	12572	13032	13527	13745	14254	14758	15425	15916	16991	17758	18631	19618	20396
ppi2 1-80µM-2	12960	13300	13895	14015	14573	15139	15204	15717	16200	17389	18096	18415	19622	20444	21311
ppi2 1-80µM-3	11292	11482	11867	12099	12695	13207	13441	13745	14141	14854	15736	15943	17147	17825	18404
ppi2 2-80µM-1	12138	12421	12774	12986	13180	13979	14120	14481	15049	15767	16576	17064	17913	18615	19287
ppi2 2-80µM-2	12940	12985	13549	13696	14289	14937	15397	15574	16172	16602	17457	18106	18736	19729	20427
ppi2 2-80µM-3	12458	13092	13252	13354	14047	14277	14536	15097	15504	15937	16709	17157	18072	19042	19592
ppi2 3-80µM-1	12100	12225	12260	12804	12861	13358	13658	13900	14104	14624	14854	15362	15826	16248	16851
ppi2 3-80µM-2	12133	12442	12747	12648	13058	13495	13633	13857	14077	14573	15086	15653	15994	16481	16922
ppi2 3-80µM-3	12016	11900	12314	12499	12798	13206	13566	13656	13875	14213	14744	15182	15554	16022	16615
blank1B	916	918	897	911	895	915	912	902	910	890	914	907	896	898	890
xrpn8a 1-0µM-1	20950	21870	23312	24892	26375	27950	30335	32618	34721	37120	40549	42257	45144	46705	50204
xrpn8a 1-0µM-2	19664	20463	21379	22397	23765	25289	26997	29096	30496	32887	35410	37707	39847	41546	43878
xrpn8a 1-0µM-3	20246	21359	22242	23543	24624	26049	27893	29660	31032	33733	35915	37692	40387	42637	44919
xrpn8a 2-0µM-1	16944	17806	18828	19730	21333	22573	24061	25678	27054	29600	31397	33794	36228	38526	40575
xrpn8a 2-0µM-2	20033	21116	21871	23190	24380	25705	27839	29460	30968	33497	35574	37652	39763	42052	45072
xrpn8a 2-0µM-3	19620	20522	21595	22787	23846	25721	26660	28730	30288	32525	34972	36779	39265	41785	43576
xrpn8a 3-0µM-1	18310	19375	20098	20906	22110	23115	24803	26203	27556	29567	31538	33179	35283	37335	39510
xrpn8a 3-0µM-2	18492	19575	20293	21580	23015	24114	25180	27668	28451	31024	32598	34849	37299	39671	41059
xrpn8a 3-0µM-3	18206	19414	20179	20773	22196	23374	24711	25943	27678	29269	30818	33129	35342	37271	39720
blank2B	854	853	833	838	873	838	817	844	824	852	821	817	844	821	844
xrpn8a 1-0.5µM-1	13768	14321	14949	15803	16523	17373	18503	19744	21265	22563	23995	25958	27307	28844	30852
xrpn8a 1-0.5µM-2	15256	15841	16426	17159	18091	18846	20199	20864	22011	23872	25316	26597	27874	29261	30572
xrpn8a 1-0.5µM-3	14776	15482	15842	16542	17572	17884	18951	19927	21009	22199	23678	25134	26678	28085	29556
xrpn8a 2-0.5µM-1	13399	13973	14356	15318	15850	16881	17425	18646	19945	21295	22512	23854	25559	27010	28543
xrpn8a 2-0.5µM-2	15389	15976	16599	17462	18092	19125	20142	21231	22047	23868	25119	26515	28056	29657	31414
xrpn8a 2-0.5µM-3	13551	14091	14569	15310	15771	16684	17730	18424	19271	20391	21892	23237	24556	25832	27461
xrpn8a 3-0.5µM-1	14856	15011	15788	16445	17009	17896	18383	19635	20592	21504	22436	23613	24963	26058	27383
xrpn8a 3-0.5µM-2	15677	16317	16849	17095	18075	18697	19515	20270	21058	22136	23348	24400	25662	26689	28214
xrpn8a 3-0.5µM-3	15282	15908	16081	16966	17619	18088	19431	19624	21054	21965	22992	24368	25795	27191	28516
blank3B	1832	1871	1833	1807	1761	1829	1823	1855	1856	1811	1843	1806	1827	1816	1798
xrpn8a 1-2µM-1	14281	14726	15284	16181	16972	17813	18798	19607	21012	22454	23820	24721	25896	27419	28892
xrpn8a 1-2µM-2	14269	14912	15224	16091	16748	17458	18464	19314	20653	21827	23299	24536	25576	26919	28508
xrpn8a 1-2µM-3	12777	13274	13762	14200	14966	15357	16372	17129	18283	19233	20574	21658	22716	23889	25372
xrpn8a 2-2µM-1	12978	13410	13892	14252	15112	15980	16296	17220	17947	19077	20413	21182	22431	24007	25189
xrpn8a 2-2µM-2	14987	15489	16353	16703	17393	18021	18738	19752	20956	22343	23263	24665	26197	27361	28737
xrpn8a 2-2µM-3	13635	14055	14895	15282	16059	16863	17398	18352	19226	20327	21725	22667	24273	25389	26743
xrpn8a 3-2µM-1	13830	14395	14811	15216	15983	16567	17037	17836	18465	19576	20308	21499	22245	23140	24312
xrpn8a 3-2µM-2	14254	14630	14955	15464	16033	16754	17134	17849	18339	19461	20457	21266	22510	23519	24325
xrpn8a 3-2µM-3	13500	13670	14288	14719	15296	15584	16191	16775	17526	18436	19284	20279	21361	22392	23131
blank	782	759	765	752	773	750	745	734	744	742	743	725	757	745	738
xrpn8a 1-80µM-1	11779	12057	12434	13068	13516	14024	14380	14985	15848	16718	17553	18420	19335	20592	21422
xrpn8a 1-80µM-2	12084	12470	12658	12987	13490	13823	14200	14929	15593	16457	17239	17926	18854	19785	20635
xrpn8a 1-80µM-3	11680	11673	12315	12795	13045	13550	14185	14615	15166	15915	16752	17497	18329	18938	19792
xrpn8a 2-80µM-1	13591	13945	14479	14752	15502	15815	16368	17119	17762	18690	19349	20342	21314	22334	23056
xrpn8a 2-80µM-2	11603	11867	12150	12433	13144	13583	13995	14419	15105	15988	16822	17164	18254	19253	20366
xrpn8a 2-80µM-3	12419	12636	13298	13471	13885	14276	15062	15661	16449	17197	17813	18663	19548	20436	21360
xrpn8a 3-80µM-1	11895	12162	12649	12819	13245	13526	13886	14563	15039	15254	15935	16594	17033	17815	18576
xrpn8a 3-80µM-2	12174	12481	12732	12931	13525	13888	14215	14499	15239	15728	16461	16750	17406	18043	18838
xrpn8a 3-80µM-3	12332	12552	12945	13321	13683	14192	14285	14921	15272	16004	16213	17074	17764	18533	19249
leer	78	76	79	68	77	78	73	74	75	74	69	78	69	70	70

Fig.06

Proteasome activity assay (AMC assay) of three biological replicates and three technical replicates, Excitation 360 nm, Emission 440 nm, 126 - 248 min

time [min]	126.30	130.65	135.01	139.36	143.73	148.10	152.51	156.91	161.30	165.68	170.06	174.41	178.78	183.14	187.49	191.82	196.16	200.50	204.81	209.14	213.45	217.75	222.06	226.38	230.70	235.01	239.31	243.63	247.93
ppi2 1-0µM-1	46956	49452	51091	54602	56360	59224	61991	64075	67381	68817	72650	74143	76753	77775	81200	84949	86530	87328	89264	93677	95312	96141	98957	101227	103091	105383	108990	111797	111574
ppi2 1-0µM-2	47452	50434	52579	55380	56324	59366	62454	64789	67883	70311	73576	74908	77616	77607	82249	85511	86763	87288	91840	94333	97437	98151	101249	101774	103247	107075	109648	112869	114779
ppi2 1-0µM-3	43857	47000	49377	50836	54273	56438	58618	61694	63785	66963	68703	70209	73844	75922	78349	80436	81979	84978	86561	88946	91198	93659	94243	96948	99818	100919	103754	106709	107394
ppi2 2-0µM-1	43807	46530	49014	51005	53765	56805	59498	61753	64154	65789	68204	72199	74188	75703	77860	80734	83050	86341	87069	89845	92910	94026	96554	98246	100921	102870	104452	107126	111330
ppi2 2-0µM-2	43814	46049	48394	50779	53084	55855	59788	60827	64202	66620	68530	71343	73845	76146	78443	81124	82287	84596	87827	89693	93247	92574	96071	99096	100575	101548	104615	107259	109618
ppi2 2-0µM-3	43711	46296	48839	51041	53448	55968	58321	61004	63196	65511	68688	69728	73110	75897	78169	80352	81047	84968	85981	88489	89975	93387	93795	96068	98300	100140	102459	104062	106804
ppi2 3-0µM-1	43109	44840	46585	48802	50491	52169	54611	56304	58270	61057	62099	64810	66584	68462	69668	72018	73165	74438	77290	79179	80829	81521	83071	85091	86650	89355	91232	92486	94585
ppi2 3-0µM-2	43593	45333	47548	49242	50929	52926	55309	56874	59861	61801	63309	65709	67027	68503	70694	72858	75541	75265	78349	79586	82363	82486	84433	86995	88182	89595	90537	94924	95322
ppi2 3-0µM-3	38268	40202	42415	44337	46618	48801	51710	54033	56052	57348	59535	61941	63577	66140	68588	70025	71024	73711	75888	78212	80008	82172	83819	85348	87303	89427	91586	93834	95146
blank1A	12740	12787	13032	13146	13301	13700	13815	14299	14400	14516	14979	15063	15349	15402	15777	15981	16304	16265	16747	16879	16992	17169	17317	17644	17645	18043	18156	18502	18724
ppi2 1-0.5µM-1	31470	32867	34121	36042	37691	39095	41342	42312	44207	45687	46894	48760	50811	51817	53241	54375	56295	58185	58852	60828	62706	62876	65185	66613	68906	69058	71675	72979	74361
ppi2 1-0.5µM-2	32588	34404	35978	37099	38696	40357	42259	43041	45208	46525	47478	49394	50681	52673	54169	55064	56804	59054	60424	61290	61980	63701	65593	67290	68340	69634	71325	72615	74193
ppi2 1-0.5µM-3	31427	33242	34632	36331	38201	39489	42042	43750	45095	46837	47959	49196	50951	53281	54108	56701	57534	59214	60276	62579	63536	65144	67264	68190	69981	71248	73905	76141	75042
ppi2 2-0.5µM-1	26355	27303	28991	30351	31807	33748	35037	35771	38277	38939	39918	41643	43341	45074	45941	47148	48846	49543	51148	52914	54566	54416	55972	57880	58138	60224	62311	62974	65043
ppi2 2-0.5µM-2	27559	28884	30824	32549	33668	35766	36919	38861	40605	41788	43142	44808	46065	47509	49080	51174	51842	53134	55327	57363	57779	59453	61396	62012	63710	64210	66850	68729	69796
ppi2 2-0.5µM-3	28134	29496	31420	32821	34393	35885	37813	39636	41283	42404	43544	46099	47779	49079	50022	52112	52592	54149	56015	56893	57887	60005	61359	62792	64089	66174	67173	67597	70085
ppi2 3-0.5µM-1	21940	23195	24309	25432	26337	27534	29265	30407	31705	32741	33622	34901	36267	37428	37898	39662	40577	41084	42584	43556	45392	46378	46737	47964	49038	50752	51398	52607	54000
ppi2 3-0.5µM-2	25637	26874	27830	28347	29713	30560	32236	33392	34081	35138	36802	37660	37902	39886	40897	41134	42435	43876	44804	46308	46829	47592	47875	48896	49687	52141	52644	53812	55383
ppi2 3-0.5µM-3	23448	24559	25647	26614	27888	29519	30537	31831	33121	33963	35666	35852	37750	38708	39600	41130	42788	43731	44890	45588	47465	47753	49038	50215	51054	52698	53781	54767	56441
blank2A	12964	13171	13316	13381	13715	13847	14318	14617	14852	14927	15200	15458	15901	16017	15898	16505	16633	16325	16865	17386	17314	17268	17757	17817	18101	18194	18554	18910	19185
ppi2 1-2µM-1	26580	27817	29407	30723	31447	32845	34298	36246	37318	38537	40173	40769	41931	43942	44644	45887	47035	48274	49895	50825	53452	53421	53733	55920	56903	58364	59787	60016	60888
ppi2 1-2µM-2	25813	26867	28252	28965	30261	31669	33074	34766	36215	36876	38415	39594	40015	42034	43324	44027	45093	46751	47718	48976	49790	50969	52398	53046	54400	56055	57564	58334	59027
ppi2 1-2µM-3	26281	27801	28586	30439	31019	32935	34239	35907	36473	38418	39223	40312	41967	43016	43850	45522	46755	47841	48894	50587	50869	51807	53552	56010	56905	57314	59147	59757	61157
ppi2 2-2µM-1	24739	26135	27055	28253	29494	30790	31647	33559	34444	35616	36983	38075	39749	40716	42160	43063	44975	45539	46494	47606	49519	50266	50496	52718	53274	53743	55997	57274	58011
ppi2 2-2µM-2	23480	24861	25704	27429	28595	29627	30451	32692	33174	34934	36084	36775	38663	39350	40681	41957	43384	44096	46384	47263	48655	48609	50440	51396	52656	53197	54979	57070	57675
ppi2 2-2µM-3	25481	26562	27926	28940	30684	31529	33593	34641	35801	37091	38273	39211	40309	42009	43902	44468	45510	47124	47825	49577	50582	50930	52274	53219	55064	56648	58876	58136	60403
ppi2 3-2µM-1	21223	22018	22780	23620	24498	25830	26277	27628	28590	29321	30162	30648	31648	32876	33478	34854	35479	36069	36973	37961	38706	39746	40573	41112	43074	43067	44507	45505	45652
ppi2 3-2µM-2	21521	22375	22964	23872	24797	25417	26118	27568	28344	29641	30139	31235	31590	33064	33435	34524	35613	36406	36762	37876	38299	39167	39143	40815	41443	42127	43979	44324	45436
ppi2 3-2µM-3	20765	21997	22613	24068	24802	25819	27124	27441	28721	29472	30511	31494	32414	33867	34295	35327	36391	36773	37580	38930	40008	40338	41492	42347	43150	44563	45346	46483	46735
blank3A	13090	13210	13321	13433	13783	14243	14399	14735	14915	14984	15305	15544	15566	15878	16308	16272	16572	17158	17096	17392	17576	17610	17861	18039	18350	18687	18609	19196	19087
ppi2 1-80µM-1	20946	22315	22903	24073	24767	25628	26846	28039	28710	29494	30781	31978	32405	33510	34070	35377	35674	37604	38675	39515	40387	40465	41687	43618	44203	44727	45823	46937	48177
ppi2 1-80µM-2	21978	23197	23681	24498	25561	26460	27948	28442	29474	30649	31593	32348	33534	34774	35347	36218	36147	38029	38813	40104	40694	41387	42424	42790	44555	44959	45970	47678	48352
ppi2 1-80µM-3	19393	20242	21173	21826	22864	23932	25022	25734	26762	27695	28598	29276	30442	31397	32246	33625	33655	34637	36329	36988	37764	38745	39371	40322	41556	42348	42796	44114	45504
ppi2 2-80µM-1	20022	20593	21915	22723	23724	24354	25527	26548	27427	28126	29303	30412	31143	31866	33573	33421	34705	35761	37195	38125	39440	39127	40527	41330	42000	42535	43977	44967	45828
ppi2 2-80µM-2	21340	22283	23145	24012	25012	25832	26600	27667	28701	29610	30415	31719	32183	33397	34585	35100	35815	36813	37942	38872	39205	40103	41265	42271	43271	44457	44716	45660	46738
ppi2 2-80µM-3	20293	21404	22069	23329	23674	24854	25739	26906	27985	29086	29903	30212	31290	32345	33381	34108	34417	35662	36996	37877	39036	39762	40864	41207	41742	43160	43767	45283	45852
ppi2 3-80µM-1	17362	18077	18482	19259	20025	20502	21122	22335	22891	23490	24014	24160	25182	26087	26574	27649	27902	28466	29341	30517	30606	31251	32347	33113	33376	34113	34902	35889	36201
ppi2 3-80µM-2	17606	18090	18775	19141	20178	21116	21749	22235	22904	23689	24149	24950	25686	26458	27210	27797	28377	29227	29439	30598	31023	31624	31982	32850	33153	34080	35106	36372	36492
ppi2 3-80µM-3	17059	17784	18622	19168	19919	20754	21431	22132	22793	23469	24030	24659	25924	26035	27042	27578	28383	29372	30506	30459	31033	31994	32792	33827	33586	34377	35304	36484	37266
blank1B	888	894	877	869	882	917	909	920	928	926	921	934	914	925	915	900	933	890	950	925	933	921	923	890	924	921	915	927	926
xrpn8a 1-0µM-1	51696	53773	55012	58506	62081	64868	67847	69143	73337	73121	76960	78500	80188	84969	86589	88289	90367	92440	94557	97831	98915	101110	102851	105355	107249	108974	109972	114249	116125
xrpn8a 1-0µM-2	45110	48254	49522	52611	54130	57603	59858	60903	64265	65849	69007	70874	71492	75193	77050	79281	80319	82005	83896	87293	90149	88975	92600	94346	96428	98135	100790	102435	104227
xrpn8a 1-0µM-3	46284	48660	50088	53498	56379	58935	61480	63895	66370	68162	69430	73166	74294	77299	78504	82059	84056	84178	87115	90509	92550	93361	97068	98676	99904	102122	104418	107472	109501
xrpn8a 2-0µM-1	43344	45586	46943	50068	53346	55468	58991	60977	63571	65802	68820	70322	71625	74972	77959	79678	81210	84000	87326	89640	91682	93609	95139	95587	99436	100275	103613	106130	107595
xrpn8a 2-0µM-2	46230	49111	50831	53866	56550	59455	61203	65098	66541	68800	72346	73532	74192	78314	81778	83094	84260	86844	90484	92573	94359	95033	98309	101017	101476	104963	108383	108783	110099
xrpn8a 2-0µM-3	46378	48217	50854	52852	55911	58377	60757	64489	65828	68556	70495	73709	73771	77584	80044	82649	84037	85779	88664	91991	93534	94635	97131	99107	101534	103828	106274	108642	111855
xrpn8a 3-0µM-1	41966	43692	46101	47936	50057	52496	54825	56191	59089	62243	63524	65471	66766	68678	71184	73302	74779	77442	81263	81283	84263	83606	87236	89050	89044	92905	94316	96130	97877
xrpn8a 3-0µM-2	43052	45632	47666	49294	52105	54274	56603	58876	60600	62903	65427	67400	68468	72120	74080	75641	75915	79358	81942	83775	87398	86108	89569	90483	93058	95272	97606	99225	102355
xrpn8a 3-0µM-3	41371	43237	45306	47856	50126	52399	55285	57639	59129	61794	63827	65581	66530	68944	72751	73539	74847	76324	79829	82529	86139	84570	86726	88488	91666	93887	95209	97963	100184
blank2B	860	838	813	805	841	810	799	810	802	828	821	805	776	836	800	822	812	806	824	809	822	823	814	842	834	867	862	832	860
xrpn8a 1-0.5µM-1	32306	33739	34972	36935	38489	39884	42636	44183	46078	47641	48960	50595	50970	54048	54927	56737	56898	59916	61656	63230	64084	65572	67435	68008	70946	73141	73195	74833	76446
xrpn8a 1-0.5µM-2	32785	34387	35803	37067	39212	40244	42596	44197	46360	47295	48374	50294	50937	53854	54236	56541	57196	58966	61283	63683	63009	64659	66696	67891	69663	70841	73259	74563	75061
xrpn8a 1-0.5µM-3	31708	33170	34432	35898	36930	39042	40323	41751	43624	45803	46927	48412	49091	52150	53351	53932	55346	57905	58903	61667	62089	63738	64816	66654	67697	68504	70817	72440	72646
xrpn8a 2-0.5µM-1	30696	31794	34130	35808	37647	39008	40935	43054	43944	46305	48431	49140	50880	53457	54904	56864	57088	59743	61978	62121	66082	66900	67212	68693	71316	73611	75698	76323	77615
xrpn8a 2-0.5µM-2	33009	34978	36614	38665	39991	41898	43922	46010	47183	49156	51286	52624	52984	56346	57468	59617	59926	62846	65545	66182	68765	69062	70379	73455	74312	73895	76808	79475	80635
xrpn8a 2-0.5µM-3	28998	30585	32365	34325	36035	37652	39337	41308	42657	44694	46731	47311	49096	50649	53007	54660	56272	57902	60294	61023	64285	63539	66804	67353	68925	71500	72382	73560	74917
xrpn8a 3-0.5µM-1	28553	30044	31114	32969	34456	35568	37310	38877	39830	41679	42063	44041	43996	46225	47603	49615	50698	52660	52651	54548	55921	57250	57900	59974	60871	61862	65038	65330	65946
xrpn8a 3-0.5µM-2	29957	30235	32230	33828	35067	36390	38202	39117	40823	42106	43508	44836	45411	47888	48906	49892	50927	52292	54120	55957	57585	58030	59514	61028	62437	63713	66167	68307	68233
xrpn8a 3-0.5µM-3	29510	31105	32100	33644	35482	36725	38654	39671	41485	43386	44598	45579	46589	48964	48981	51499	51308	52651	55206	56982	58883	58903	60465	61924	63471	64955	67072	66617	69982
blank3B	1844	1805	1782	1781	1822	1803	1816	1782	1854	1795	1801	1800	1763	1830	1803	1815	1783	1793	1828	1822	1810	1811	1789	1830	1853	1808	1849	1864	1850
xrpn8a 1-2µM-1	30155	31705	32903	34508	35909	37557	39780	40271	41371	42869	44264	45821	45906	48457	49784	50707	51458	53616	55255	56842	57471	58508	60292	60888	62027	65109	66361	66587	68624
xrpn8a 1-2µM-2	29933	30928	32323	33360	35077	36182	37904	38826	40465	41600	42547	43949	44913	46749	48343	49289	49899	52370	53255	54194	55122	56480	57907	59350	60618	61618	63239	65069	65983
xrpn8a 1-2µM-3	26573	27991	29501	30456	31842	33101	34924	35612	37313	38900	39637	41276	41944	42945	44752	45940	47036	49105	49957	51389	52840	53117	54950	56123	57447	58031	59675	61445	62886
xrpn8a 2-2µM-1	26576	27650	29494	31329	32363	34042	35360	37027	38472	39569	41098	42787	43136	46311	47563	48698	49933	51655	52603	54041	54998	56716	58418	59078	61011	62145	64039	65232	67695
xrpn8a 2-2µM-2	29956	32007	32822	34482	35651	37862	39554	41269	42208	44118	44360	46143	47457	49902	51482	51870	53482	55267	57438	58080	59620	60616	62662	62886	65129	67189	68285	69521	70682
xrpn8a 2-2µM-3	28256	29369	30982	32868	33965	35339	37531	39139	40700	42119	42732	44244	45430	47673	49405	50526	51046	53817	54479	56078	57744	58527	60112	61815	62683	64996	66323	68379	68735
xrpn8a 3-2µM-1	25696	26554	27595	29039	29887	31617	32075	33385	34773	36110	36723	37583	38317	39861	41332	42118	42779	44685	45291	46726	46731	48340	49416	50701	51895	53444	53963	55537	56381
xrpn8a 3-2µM-2	25822	26761	27556	28789	29901	31268	32326	33272	34426	36272	36988	37956	38507	40326	41354	42716	42741	44893	46081	46566	47628	48595	49394	51011	52483	53218	55009	55764	56497
xrpn8a 3-2µM-3	24380	25432	26500	27648	28513	30133	31601	32868	33554	35025	36005	37104	37490	38902	40224	41776	42253	44110	44458	46456	47358	48402	49564	50228	51739	52572	53800	54868	56172
blank	725	738	742	729	716	747	739	728	721	717	716	719	716	716	739	725	747	746	705	764	730	725	712	757	711	723	723	756	724
xrpn8a 1-80µM-1	22382	23021	24126	25167	26553	27706	28872	30265	30678	31838	33089	33799	34692	36297	37529	38299	38369	40452	41299	42622	43134	43897	45214	46943	48069	48948	49641	51005	51865
xrpn8a 1-80µM-2	21865	22392	23416	24479	25516	26402	27594	29124	29267	30738	31772	32677	33263	35111	36336	36639	37274	38556	39627	40982	41568	41964	44005	44270	45715	47545	48418	49148	49877
xrpn8a 1-80µM-3	20892	21952	22902	23578	24246	26054	26975	28413	28730	29990	30496	31601	32182	33653	34541	36217	36353	38117	39181	40122	40726	40807	42491	43655	45044	45539	47079	47378	48816
xrpn8a 2-80µM-1	24392	25113	26475	27379	28418	29204	31136	31732	32633	34040	34685	36090	36224	38612	39312	40322	41037	42834	43382	45650	46518	46750	47632	49227	50098	51276	52781	53957	54624
xrpn8a 2-80µM-2	21398	22233	23064	24171	24959	25933	27180	28576	29639	30768	31567	32953	33407	34176	36274	37434	37329	39156	40280	42491	42374	43129	44912	45425	46549	47427	49562	49906	51073
xrpn8a 2-80µM-3	22136	23505	24220	25206	26319	27839	28690	29338	30842	32138	32627	34116	34412	35512	37057	37696	38681	40819	41408	42445	43290	44621	46204	46802	48195	48810	50178	51707	52514
xrpn8a 3-80µM-1	19163	19980	21146	21581	22647	23419	24230	25535	26165	27073	28254	28586	29072	30549	31571	32147	32631	34597	35134	36045	36244	37621	38833	39624	40041	41495	41769	43338	43376
xrpn8a 3-80µM-2	19600	20451	21232	22094	23294	23945	24726	25440	26454	27251	27840	29121	29597	31127	31354	32750	32706	34450	35300	36497	36981	38010	38626	39645	39844	41514	42054	42895	44346
xrpn8a 3-80µM-3	20042	21070	21784	22499	23236	23964	25385	26408	26844	28134	28878	29572	30176	31549	32728	33303	34428	35644	35839	36771	37996	38077	39335	40495	41265	42698	43335	45143	45446
leer	70	72	81	67	81	85	79	72	69	68	78	63	78	71	76	76	62	74	73	76	76	77	79	75	68	69	80	80	72

Fig.06

Proteasome activity assay (AMC assay) of three biological replicates and three technical replicates, Excitation 360 nm, Emission 440 nm, 252 – 365 min

time [min]	252.26	256.57	260.89	265.20	269.50	273.84	278.17	282.52	286.86	291.17	295.51	299.86	304.20	308.51	312.84	317.19	321.55	325.89	330.21	334.56	338.89	343.22	347.51	351.82	356.16	360.46	364.77
ppi2 1-0µM-1	114747	116593	117827	120001	124212	126254	127151	130039	128951	132556	134371	138112	137971	140866	144557	144563	144427	150219	151060	151537	155900	155598	157088	158578	161278	163027	163680
ppi2 1-0µM-2	116326	116907	121091	123062	125534	126595	130500	131822	132365	134282	137133	139564	141456	142602	144570	145361	148254	151294	154420	154271	158079	158174	161566	161979	162624	165006	167003
ppi2 1-0µM-3	109646	112262	114182	115791	117665	119188	121836	122848	125910	127099	129628	132314	134710	134885	137767	140668	140298	143455	146720	148944	149615	151516	153683	154327	155004	158535	159435
ppi2 2-0µM-1	111823	113876	118327	117003	120487	122373	124781	127414	128449	131482	133642	134328	137732	138285	140187	142187	142848	147887	150776	150435	151996	154401	157420	156989	159535	160184	161742
ppi2 2-0µM-2	113131	115536	116298	118127	120963	123527	126272	127027	129341	130654	131659	134381	135470	137623	141080	143970	142640	147738	148535	151872	152228	154571	157155	159351	159808	162540	162099
ppi2 2-0µM-3	109265	111674	113199	114161	117138	120149	120703	123840	125268	126427	128319	131638	134238	133276	135888	139229	137569	141227	143839	146789	147358	149016	151065	151929	153547	156345	156777
ppi2 3-0µM-1	95891	98046	99638	101683	104460	106285	107809	108584	111057	110944	113490	115379	116349	118031	118251	120322	122085	123475	126115	127368	129397	130696	132592	134914	134251	135292	137532
ppi2 3-0µM-2	97392	99800	100844	102324	105371	107001	108699	110310	110943	113154	115958	117967	119094	118865	121119	123065	122086	127788	129056	131513	131676	133565	136348	135741	136911	138691	138955
ppi2 3-0µM-3	96588	99239	100360	103092	105602	108588	108971	110327	111546	115855	114945	116566	119583	120882	122616	125103	126547	128851	131139	131994	134108	135491	137302	139296	141201	142242	143727
blank1A	18942	18942	19374	19699	19861	20215	20151	20549	20696	21020	21092	20603	21726	22272	22011	22270	22066	22647	23066	23425	23392	23588	23884	23866	24286	24321	24326
ppi2 1-0.5µM-1	75171	76627	78883	80163	81550	82523	85614	85978	87061	90286	90880	92461	93315	94767	96951	97892	99012	102200	102533	104151	104665	106203	109464	109670	110875	110735	113316
ppi2 1-0.5µM-2	76521	77351	78615	79984	81483	83784	84889	87009	86086	86694	88541	92075	92647	95024	95033	96980	97272	100059	102021	102496	103814	106414	107169	107551	109789	111303	113524
ppi2 1-0.5µM-3	78117	80268	80335	83125	85297	86601	88365	88232	89465	92434	94066	95490	97357	97832	100543	101205	101525	105580	106615	107646	109853	110215	112697	113667	114892	116576	118635
ppi2 2-0.5µM-1	65446	67762	68081	70170	72128	73022	74691	75415	77319	78678	79225	81594	82639	83575	84996	86266	87198	88540	90282	91201	92708	94379	94836	96549	96874	99574	98118
ppi2 2-0.5µM-2	70395	72372	73767	75280	77290	78107	79778	82548	83436	85433	86186	87161	87955	90567	91123	92609	92252	94984	96994	97872	100156	101436	102967	104139	105328	106758	106407
ppi2 2-0.5µM-3	72373	73903	74987	75842	78702	77425	80593	81245	83132	84817	86640	88530	89310	89733	92661	91321	93154	97157	97438	98527	100196	103302	103422	104394	104420	107784	106529
ppi2 3-0.5µM-1	54673	56028	57472	58318	60184	61471	62260	62523	64227	65415	66686	67240	68214	69660	71689	71968	72242	74224	75813	77940	78579	79978	81786	81761	83857	83462	84623
ppi2 3-0.5µM-2	55850	56607	58348	59222	60659	61890	63026	64144	64357	65365	67024	67906	68542	70200	70942	72207	72734	73806	75294	77328	77650	78759	79354	81217	81838	82204	83739
ppi2 3-0.5µM-3	57471	58235	60461	60775	63015	64134	64641	66580	67149	67815	69662	69460	71563	73663	73306	75346	75556	78475	78081	80319	81341	82389	84278	84864	84259	86681	87595
blank2A	19244	19708	19583	20087	20103	20494	20484	21081	20623	21124	21398	21514	21493	22158	22093	22187	22278	22861	23191	23457	23627	23344	23843	24106	23949	24620	24846
ppi2 1-2µM-1	62668	64804	65637	66530	68507	70144	70474	72043	72545	74265	75457	77514	76916	78788	79130	81241	81688	84672	85813	85971	86716	88745	90058	91401	91176	94018	95029
ppi2 1-2µM-2	61533	61988	63080	64393	66014	66605	68057	69284	70963	71448	72478	73789	74715	76187	77292	78830	78933	81265	82383	82818	84385	84661	88244	89228	87477	89865	89732
ppi2 1-2µM-3	62387	64241	64735	65927	68285	68757	70740	71617	72629	74688	75461	76655	77214	78395	80408	81419	82162	83687	85268	85710	87185	88838	90995	90473	91924	93642	94546
ppi2 2-2µM-1	59906	60467	62087	63414	63896	65782	66416	67686	68354	69707	70752	72347	72658	74361	75182	77177	78169	78698	80761	81328	81702	83161	85300	85990	87538	87421	88536
ppi2 2-2µM-2	58396	60297	61386	62123	63903	65002	66096	66630	67438	68918	71017	72511	73285	73983	76070	76361	77645	79905	81054	81956	83074	83426	86188	87711	87810	88112	88813
ppi2 2-2µM-3	61443	62373	63870	64565	66693	67785	69004	69506	71993	72036	73059	75138	76412	75738	77838	79587	80526	81562	83070	83842	84957	87278	88314	87755	89419	90572	90264
ppi2 3-2µM-1	47118	48370	48958	49763	50876	51563	53376	53748	54592	54484	56310	57045	57163	59032	59456	60746	61254	62156	64591	64909	64555	66078	67344	67191	68325	69500	70259
ppi2 3-2µM-2	46522	47107	48087	49232	49815	51271	51966	52438	53827	54586	54871	56253	56307	57723	58636	59068	59330	61204	62535	62896	63497	65653	65315	66274	66554	68953	67741
ppi2 3-2µM-3	47587	49134	49567	51608	52270	53280	53566	54946	55498	56525	57233	58508	59851	60446	61553	61555	62434	64174	65304	67340	67032	68701	70944	70246	71101	72293	73245
blank3A	19669	19809	19775	20233	20698	20735	20921	20952	20855	21453	21718	21749	22059	22324	22545	22489	22628	23102	23471	23652	23858	23848	24609	24665	24833	24929	24657
ppi2 1-80µM-1	49326	49513	51422	51915	52939	54683	55830	56701	57100	58723	58905	59908	60667	61968	63248	63399	64934	65399	67579	67829	69308	70820	70723	72013	72027	72864	73358
ppi2 1-80µM-2	48528	50011	50461	51915	53499	54036	54753	55912	56582	57274	58657	58518	60315	60549	62541	63194	62686	65291	66816	67423	67571	69019	69228	71327	71383	72287	72553
ppi2 1-80µM-3	45847	47487	47895	48374	50699	50564	51262	52683	54011	54383	55672	56357	57002	58291	58669	59509	59942	61746	62926	65171	64352	65567	66430	67760	68358	68611	69559
ppi2 2-80µM-1	46725	48580	48781	49364	51133	52224	52975	53952	55162	55356	55809	56202	57667	58971	59977	59969	61548	63007	64153	65769	65140	67619	67102	67986	69203	68510	70017
ppi2 2-80µM-2	48385	49059	49793	51700	51665	52148	54121	54873	55544	56026	58110	57900	59090	60194	60180	61684	62165	63337	64423	65112	66647	67177	68372	68083	69443	70159	70785
ppi2 2-80µM-3	47114	48056	49415	50291	51546	52196	52396	53999	54467	55233	55781	56430	58742	58579	59767	60536	62029	63475	64286	64477	64816	65779	67558	68601	69543	70064	70857
ppi2 3-80µM-1	37114	38056	38572	38967	40059	41551	41776	41917	42448	43491	44347	44680	45505	46251	46971	46921	48085	49170	50240	50904	52100	52605	53472	54143	54476	55331	55338
ppi2 3-80µM-2	37533	38314	39532	39474	40447	41537	41878	42042	43191	43766	45250	45105	46140	46516	46688	47337	48588	49878	50496	51398	52248	52604	53705	54086	55070	55707	55985
ppi2 3-80µM-3	37580	38243	39489	39830	41270	41499	42476	43224	43523	44815	45307	46220	46650	46906	47575	49252	48963	49960	51774	52689	52612	53199	54624	55389	56004	56033	56793
blank1B	920	956	970	931	951	908	946	935	933	918	954	967	948	923	932	920	957	936	922	971	978	949	950	961	957	943	956
xrpn8a 1-0µM-1	117583	118844	123551	125723	126203	128921	131557	132226	133364	135623	137555	139005	143004	143568	145759	147566	147554	150647	154771	155188	155763	156878	158380	161676	163115	162051	165917
xrpn8a 1-0µM-2	104912	108315	110684	111722	114900	115829	117015	119311	121611	123061	123091	127452	127961	131536	131942	132060	132718	138299	138667	140738	141596	143056	149325	146358	148564	148089	148909
xrpn8a 1-0µM-3	110265	113271	115072	118230	120752	121399	123504	123040	127473	127984	129502	133374	132103	136312	136112	138176	138309	141452	144444	145693	148230	149252	151181	152639	152816	154198	156933
xrpn8a 2-0µM-1	110287	113072	114888	116637	119391	122113	123854	126540	126167	130509	131338	133410	133280	137386	139808	140527	143448	142742	146689	148565	150530	152119	153806	154920	157513	157917	162604
xrpn8a 2-0µM-2	113871	116501	118059	120338	119622	123185	125314	128476	129665	132711	134202	134892	138342	137776	141455	139520	145050	146428	149131	150487	151869	154357	155207	155990	157164	157910	160687
xrpn8a 2-0µM-3	112322	114116	115746	120777	122344	122253	125658	126605	129281	131451	131665	134215	137206	137485	139445	140474	145651	147041	148721	149589	150263	155297	156907	155824	158080	156313	161404
xrpn8a 3-0µM-1	99219	103416	106339	105298	109324	111416	112188	113681	118064	115971	119836	121995	122806	122759	125561	127299	130476	131881	133172	136032	136882	140491	139828	141558	142634	142899	147985
xrpn8a 3-0µM-2	101161	106026	108076	110374	112256	111723	115175	115384	117062	119620	120168	121212	124003	127512	127529	129930	129824	135277	136581	137866	140224	142131	143680	143763	143948	147052	147257
xrpn8a 3-0µM-3	100560	104247	105776	108250	109982	111294	113847	115237	118594	119301	119863	122010	123169	123725	127176	127190	132145	132915	135959	136441	138250	139853	143218	142889	146184	146826	147495
blank2B	837	869	836	877	878	885	871	874	852	841	856	866	860	850	829	841	827	858	858	889	867	855	867	908	879	872	880
xrpn8a 1-0.5µM-1	77877	80457	82325	83955	85153	87318	87901	90149	90121	93188	93719	95626	95477	97415	98857	98872	102014	104372	106634	106257	107335	110935	113853	113666	114480	114666	117214
xrpn8a 1-0.5µM-2	75729	79203	80484	81768	84076	84612	86320	87324	89428	89062	91191	93665	94268	96702	97017	99037	101301	101296	103026	104484	105490	106760	107796	110581	110297	114070	113259
xrpn8a 1-0.5µM-3	74813	77958	79561	81484	81380	82314	85676	85040	87596	88920	90026	89331	91343	94563	95367	96639	98747	99125	102049	102805	104380	105892	106115	107876	109524	111224	112295
xrpn8a 2-0.5µM-1	78485	81615	84013	84265	87003	87757	89442	92207	92607	94044	96378	97321	97967	99498	102059	103559	105462	106815	109076	111292	110783	113657	116154	116722	116229	119330	120222
xrpn8a 2-0.5µM-2	81203	84457	85485	88221	89572	91812	93235	93313	94698	97059	97721	99590	100966	103836	103229	105412	108845	110437	110290	113022	114941	116169	118133	118743	120450	121890	123268
xrpn8a 2-0.5µM-3	76723	79830	81152	82291	84529	86927	87085	88200	90150	92294	94235	94386	95165	96514	99960	102366	100787	105586	106774	107853	108413	111440	110750	113439	113929	116161	118007
xrpn8a 3-0.5µM-1	68482	69982	70751	73309	73974	74961	75493	78329	78817	80465	81975	83380	83418	87160	85863	87847	89918	91896	92008	93808	95664	96476	98529	99647	101478	101685	103413
xrpn8a 3-0.5µM-2	69238	71711	72516	73927	75495	76290	77321	79585	81586	82048	82975	84275	85282	87675	89365	88827	91524	93665	94923	96349	98500	99371	99949	100963	102508	104672	104879
xrpn8a 3-0.5µM-3	70572	71832	73551	74956	77952	78803	79721	80274	83004	84062	85542	85757	87203	88599	90506	91140	93101	95361	97460	98108	100341	100724	102552	104270	104772	104582	107251
blank3B	1828	1829	1823	1839	1893	1899	1861	1836	1842	1893	1889	1882	1850	1872	1796	1862	1900	1874	1831	1859	1873	1829	1905	1896	1901	1830	1860
xrpn8a 1-2µM-1	70288	71022	73016	74294	76088	75824	78347	78643	80707	83091	83458	84477	83861	86196	88392	88523	90267	92072	92842	95273	96627	96542	97917	99343	100560	101231	101363
xrpn8a 1-2µM-2	65695	68274	70076	71003	72805	73229	75238	76421	76298	79089	79161	80396	81672	82797	84844	84968	87591	87771	89761	90460	91422	92307	94179	95049	96108	96786	98187
xrpn8a 1-2µM-3	63756	64321	66616	67394	70131	70778	71716	72160	74819	75297	75338	77885	78680	80615	80965	82678	84558	85150	85823	87337	88120	89653	91930	92430	94672	95039	94991
xrpn8a 2-2µM-1	67539	69994	71677	72812	75007	74170	77304	78087	79297	80855	82507	83482	84215	85089	86864	88218	90058	92193	94059	95264	96849	97412	97348	98988	101478	102176	102492
xrpn8a 2-2µM-2	72680	74984	75224	76796	77871	79330	80511	81681	84042	86568	85492	88174	87783	89974	92806	91138	93711	96725	98576	99022	100127	102689	101190	102464	104520	107428	107929
xrpn8a 2-2µM-3	68821	71628	72950	74750	77175	77390	80181	80197	81713	82306	84113	86655	86844	87586	89805	89821	92194	93551	94698	95691	98292	99349	99754	101961	103565	103946	105634
xrpn8a 3-2µM-1	56967	58682	59386	61234	62507	63941	65198	65992	66776	68602	69319	70596	70673	71345	73098	73082	74945	76915	78480	79448	80377	81827	82793	82733	84961	86075	86504
xrpn8a 3-2µM-2	57152	59934	61118	62504	62612	63945	65308	65534	66485	68174	69058	70606	71002	72446	74531	74762	75705	77724	79331	79479	80467	82699	83245	83852	85564	85657	87331
xrpn8a 3-2µM-3	57281	58489	59955	61633	62047	64024	64138	65332	66613	67855	68812	70531	71150	73047	74021	74024	75390	77210	78492	79563	80400	82561	83081	82991	85775	86117	87601
blank	730	747	758	760	745	728	723	738	744	722	735	728	750	745	719	762	728	721	744	728	731	745	751	730	761	761	738
xrpn8a 1-80µM-1	53466	54641	55475	56628	57344	59415	60397	59776	61603	63095	62511	64860	66029	67364	67232	68865	69787	70963	72998	74587	74752	76380	75591	77510	78624	78897	79920
xrpn8a 1-80µM-2	50601	52573	53479	54439	54385	56562	57466	58113	59110	60370	61658	62268	63397	63316	65273	66190	66542	68076	69416	70111	71594	71993	73327	74078	75824	76666	76588
xrpn8a 1-80µM-3	50032	51139	52453	53688	54523	55314	56243	57059	57622	59125	60117	60349	62161	62275	63994	64086	65463	67223	68369	69029	70255	70835	72704	73383	74380	75399	75251
xrpn8a 2-80µM-1	55602	57322	57907	59952	60682	61538	63289	63339	65101	65838	67183	68223	70629	69864	70633	71699	72889	74673	75426	75823	78486	78903	79830	79902	82984	83032	84262
xrpn8a 2-80µM-2	51593	53371	54069	56065	56587	58425	60012	60394	61010	62509	63563	64796	65506	66044	67725	67347	70217	70136	72076	73626	74053	75554	75394	77726	78099	78458	80304
xrpn8a 2-80µM-3	52994	54782	55831	57291	58861	59872	60229	60918	62374	63607	64738	65856	66545	67628	68831	68817	70175	70799	73416	74439	76175	77292	77954	77269	79568	79860	81605
xrpn8a 3-80µM-1	44658	46345	46798	48096	49008	49843	50145	51615	53112	53472	54608	55230	56227	56993	57587	57932	60964	60698	62284	62518	63852	65042	65782	66234	67495	68226	69348
xrpn8a 3-80µM-2	45283	46078	47316	48911	49589	50490	51384	51875	52805	53549	55035	55361	56269	57742	58562	58458	60833	61461	62960	63166	64829	64649	65968	66403	67716	68918	69565
xrpn8a 3-80µM-3	45934	47588	49255	50006	50934	51410	53482	53558	54060	55291	55918	56732	57471	58381	59612	60454	62022	63511	64129	66150	65745	66719	67847	68086	69869	70948	72009
leer	59	68	77	77	61	82	73	69	70	85	73	76	77	69	76	68	71	72	75	69	74	66	77	71	76	74	63

Fig.06

Hypocotyl measurements (Biological replicate 1-3, n=8 each)

MG132 [µM]	WT 1	WT 2	WT 3	WT 4	WT 5	WT 6	WT 7	WT 8	WT 1	WT 2	WT 3	WT 4	WT 5	WT 6	WT 7	WT 8	WT 1	WT 2	WT 3	WT 4	WT 5	WT 6	WT 7	WT 8	p Value [DMSO vs. MG132]
DMSO	5.557	3.815	4.440	9.034	7.983	7.391	6.154	8.994	6.035	4.441	9.604	6.201	7.927	9.261	4.319	5.255	7.352	6.889	6.964	8.234	5.215	4.780	5.629	8.005	
0.5	3.038	6.005	7.237	4.759	7.912	5.136	8.125	5.884	7.966	6.494	5.581	6.190	7.218	5.736	3.892	3.477	6.167	7.945	6.428	7.108	8.076	7.554	5.743	7.072	0.436
1	3.692	8.395	6.011	5.180	9.622	6.235	4.361	5.371	7.491	7.660	5.891	5.833	6.700	6.130	4.172	6.712	7.776	6.251	4.795	5.845	6.740	6.459	4.066	7.132	0.325
15	3.930	3.106	2.741	3.190	3.124	2.150	2.466	5.153	4.276	3.744	5.840	4.586	4.345	3.291	5.809	5.562	2.933	5.345	5.481	2.499	4.801	4.430	3.349	4.679	0.000
30	3.955	3.814	2.918	1.035	3.117	4.115	0.871	1.942	3.843	3.140	4.076	1.880	3.859	5.215	2.182	3.957	1.989	2.608	2.083	4.361	3.016	3.298	2.061	4.081	0.000
50	1.043	1.426	0.576	0.647	0.889	1.033	2.868	1.489	1.786	2.084	1.588	1.767	2.333	1.214	1.791	1.203	1.605	3.316	1.440	1.609	3.217	3.053	2.448	1.880	0.000
100	0.705	0.423	0.596	0.196	0.282	0.475	0.104	0.311	0.677	0.564	0.411	0.471	0.213	0.125	0.156	0.221	0.568	0.420	0.223	0.521	0.446	0.174	0.156	0.223	0.000
200	0.000	0.000	0.000	0.000	0.000	0.000	0.000	0.000	0.269	0.000	0.267	0.294	0.000	0.204	0.287	0.200	0.126	0.000	0.441	0.000	0.323	0.447	0.203	0.234	0.000

MG132 [µM]	*rpn8a 1*	*rpn8a 2*	*rpn8a 3*	*rpn8a 4*	*rpn8a 5*	*rpn8a 6*	*rpn8a 7*	*rpn8a 8*	*rpn8a 1*	*rpn8a 2*	*rpn8a 3*	*rpn8a 4*	*rpn8a 5*	*rpn8a 6*	*rpn8a 7*	*rpn8a 8*	*rpn8a 1*	*rpn8a 2*	*rpn8a 3*	*rpn8a 4*	*rpn8a 5*	*rpn8a 6*	*rpn8a 7*	*rpn8a 8*	p Value [DMSO vs. MG132]	p Value [WT vs. *rpn8a*]
DMSO	7.579	8.190	5.217	5.235	5.922	5.955	6.202	5.203	5.697	5.973	4.119	7.126	5.264	7.517	7.160	6.422	4.673	7.215	5.385	6.403	7.426	7.395	7.682	4.788		0.342
0.5	6.315	7.762	8.332	6.061	5.760	7.752	7.987	4.661	4.395	5.437	5.730	4.988	4.705	5.044	7.031	3.354	4.813	4.404	5.976	7.489	8.137	7.645	4.475	8.339	0.735	0.689
1	8.296	5.346	5.378	7.259	7.117	4.033	5.489	5.443	5.623	7.426	6.731	6.090	6.090	4.590	5.712	6.743	5.921	6.809	7.738	7.272	4.237	6.874	3.419	4.345	0.492	0.631
15	4.285	2.565	3.834	7.107	3.225	6.359	3.676	5.069	3.398	6.196	4.234	4.075	3.549	3.593	5.509	4.563	4.703	5.283	3.271	5.560	6.468	7.373	4.416	5.053	0.000	0.057
30	2.620	6.552	5.182	4.752	2.074	2.627	2.056	2.079	4.424	6.282	5.330	3.611	4.225	6.361	5.157	3.692	4.607	2.435	3.228	4.783	3.582	5.892	4.060	4.608	0.000	0.004
50	3.147	1.907	2.307	1.784	3.513	4.838	2.996	2.261	1.912	3.700	2.158	2.126	2.193	2.698	3.072	3.781	2.645	3.640	2.855	2.743	3.340	3.048	3.056	3.781	0.000	0.000
100	0.812	1.330	1.388	1.014	1.512	0.573	1.171	0.449	0.938	1.886	2.048	0.791	1.005	1.276	2.467	1.554	1.616	1.070	1.537	0.971	1.171	0.803	0.447	0.362	0.000	0.000
200	0.219	0.750	0.393	0.366	0.219	0.000	0.000	0.000	0.579	0.920	0.797	0.926	0.334	0.473	0.878	0.517	0.816	0.787	0.769	0.720	1.610	1.427	0.615	0.931	0.000	0.000

DMSO | 15 µM | 30 µM | 50 µM | 100 µM | 200 µM

WT

rpn8a

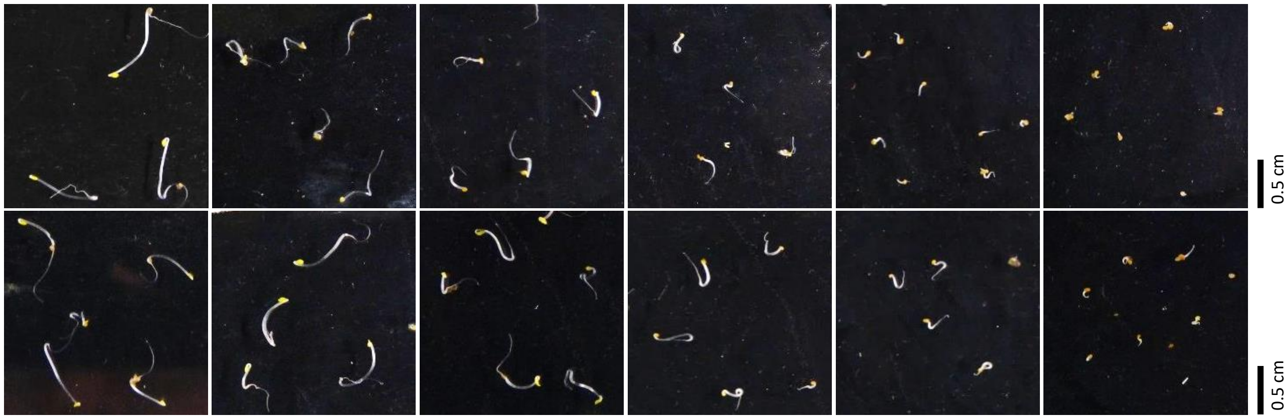

Fig.06

Real-time PCR data of proteasomal genes (3 Biological replicates, each measured in 3 technical replicates)

gene / line	ΔCt Rep 1	ΔCt Rep 2	ΔCt Rep 3	p value [**contr.** vs mut]
RPN8b		*AT3G11270*		
**WT**	**4.37**	**4.03**	**4.50**	
rpn8a	2.93	2.97	3.03	0.0007
ppi2	**3.60**	**3.57**	**3.63**	
rpn 8ax ppi2	2.27	2.60	2.50	0.0003
RPN5a		*AT5G09900*		
**WT**	**2.10**	**1.67**	**1.97**	
rpn8a	1.37	1.10	1.47	0.0236
ppi2	**1.10**	**0.93**	**0.80**	
rpn 8ax ppi2	-0.37	-0.37	-0.07	0.0008
RPN10		*AT4G38630*		
**WT**	**4.33**	**3.87**	**4.40**	
rpn8a	3.63	3.53	3.63	0.0247
ppi2	**3.72**	**3.50**	**3.80**	
rpn 8ax ppi2	2.73	2.57	2.87	0.0016
RPT2a		*AT4G29040*		
**WT**	**2.80**	**2.53**	**2.90**	
rpn8a	1.83	1.63	2.07	0.0056
ppi2	**2.37**	**2.27**	**2.47**	
rpn 8ax ppi2	1.27	1.43	1.07	0.0024
PAA2		*AT2G05840*		
**WT**	**3.30**	**3.60**	**3.63**	
rpn8a	2.40	2.50	2.53	0.0008
ppi2	**2.67**	**3.07**	**2.98**	
rpn 8ax ppi2	0.90	2.00	1.60	0.0150
CDC48a		*AT3G09840*		
**WT**	**-0.13**	**-0.20**	**0.00**	
rpn8a	-0.80	-0.70	-0.53	0.0044
ppi2	**-0.83**	**-1.00**	**-0.87**	
rpn 8ax ppi2	-1.87	-1.60	-1.67	0.0010
NAS6		*AT2G03430*		
**WT**	**5.47**	**5.57**	**6.17**	
rpn8a	4.60	4.57	4.67	0.0070
ppi2	**4.87**	**5.20**	**5.17**	
rpn 8ax ppi2	3.20	3.88	4.10	0.0098

Fig.07

Uncropped western Blots (same membrane, serial antibody detection)

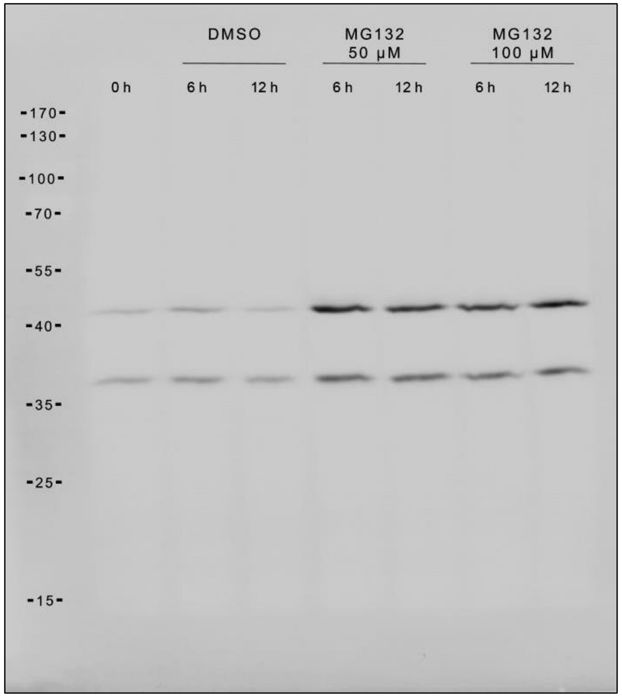

antiGFP

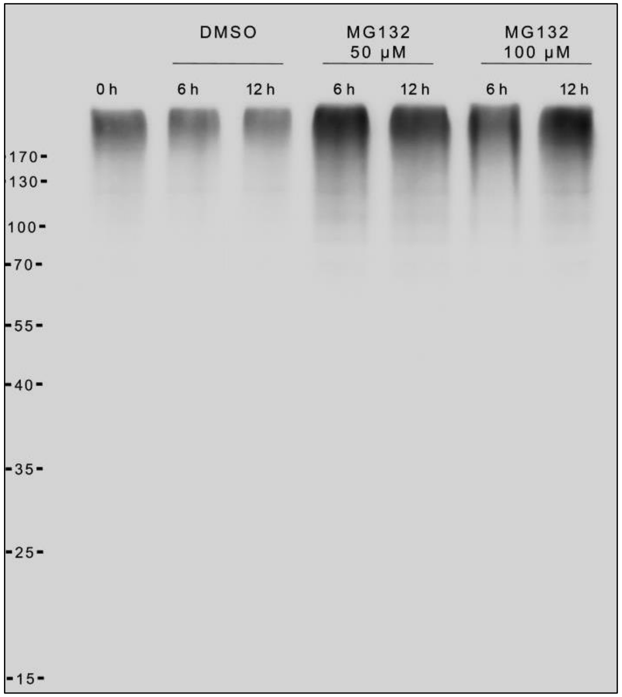

antiUbiquitin

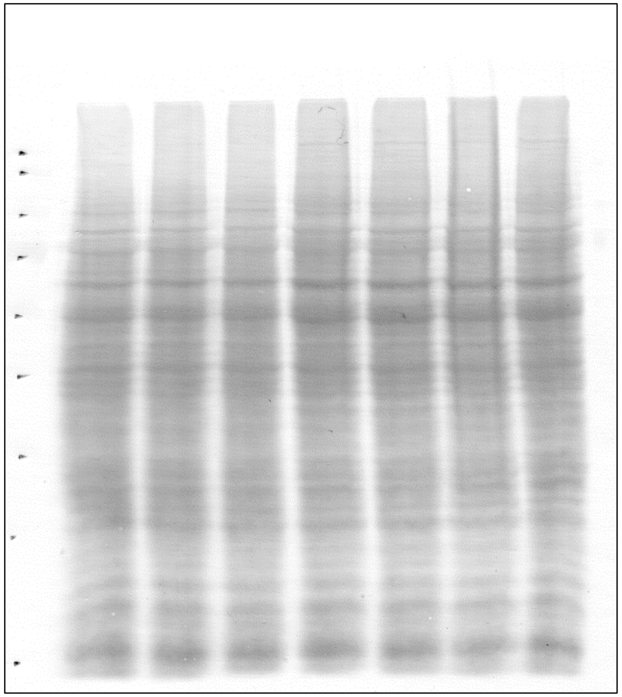

Amido Black staining

Supplementary Fig.03

Determination of fresh weight (Biological replicate 1-3, n=12 each)

	WT [mg]												*rpn8a* [mg]											
1	93.27	81.44	51.71	58.9	54.39	98.37	75.53	62.83	89.37	97.45	52.91	50.03	83.14	90.66	83.75	77.84	98.03	94.36	116.2	70.8	75.89	101.7	105.7	71.01
2	77.41	85.95	49.92	73.24	44.7	58.47	70.2	46.31	37.69	85.44	78.38	66.72	77.17	80.75	43.07	63.11	90.98	77.9	58.51	79.1	48.27	80.22	98.24	76.77
3	60.99	47.71	48.54	37.68	77.47	59.97	37.8	37.01	49.14	47.74	71.4	39.58	75.34	94.58	62.59	63.46	48.51	63.85	83.6	105.1	74.82	55.37	79.79	92.24

Determination of root length (Biological Rep 1-4; n=10 each)

		WT [cm]										*rpn8a* [cm]									
7d	1	1.177	1.702	1.08	1.459	1.463	1.261	1.501	1.365	1.016	1.056	1.341	1.303	1.81	1.595	1.778	1.721	1.255	1.754	1.744	1.491
	2	1.681	0.957	1.627	1.079	0.797	1.532	1.439	1.249	1.356	1.244	1.311	1.264	1.864	1.664	1.815	1.916	1.683	1.442	1.907	1.768
	3	1.591	1.67	1.886	2.132	1.787	1.519	1.602	1.309	1.498	1.623	1.523	1.915	1.558	1.822	1.914	1.918	2.149	2.175	2.119	1.557
	4	1.541	1.782	1.981	2.027	1.955	2.063	2.106	1.587	1.873	1.94	1.92	2.082	1.805	1.639	1.903	2.097	2.007	2.136	1.842	1.967
17d	1	9.153	9.146	7.742	8.338	8.913	9.59	9.457	9.303	8.102	7.886	7.764	7.644	8.164	7.066	8.013	8.507	7.893	8.338	7.874	7.403
	2	8.549	6.377	7.541	4.256	7.63	7.392	6.864	8.512	8.691	8.177	6.799	7.046	7.15	6.667	6.828	8.137	8.187	7.322	7.477	7.693
	3	7.424	10.446	10.531	10.299	10.424	10.742	10.225	10.074	10.084	9.865	8.65	8.118	7.643	8.645	8.662	8.279	8.805	8.775	9.174	7.289
	4	8.006	8.137	7.791	8.55	8.096	7.935	9.786	7.023	8.459	9.31	6.889	7.484	7.398	5.701	6.873	7.649	7.274	7.213	6.028	6.72

Determination of germination and greening efficiency (Biological Rep 1-3; n=50 each)

		control		+ Mannitol		+ NaCl	
		WT	*rpn8a*	WT	*rpn8a*	WT	*rpn8a*
Rep 1	#seeds	50	50	50	50	50	50
	germinated	41	47	44	46	40	44
	green	41	47	38	44	19	0
Rep2	#seeds	50	50	50	50	50	50
	germinated	37	42	42	45	40	45
	green	37	42	39	40	13	3
Rep3	#seeds	50	50	50	50	50	50
	germinated	44	46	40	47	35	35
	green	44	46	38	44	15	3

Supplementary Fig.04

Determination of pigment content (Biological replicate 1-3, n=6 each)

ppi2 NL			*rpn8a ppi2 NL*			*ppi2 LL*			*rpn8a ppi2 LL*		
**Chl a**	**Chl b**	**Carot**	**Chl a**	**Chl b**	**Carot**	**Chl a**	**Chl b**	**Carot**	**Chl a**	**Chl b**	**Carot**
5.49	2.50	5.21	10.21	4.40	7.22	13.31	6.93	6.97	18.00	8.60	9.94
5.23	3.46	4.01	13.68	7.39	10.16	14.11	7.87	7.05	23.27	12.46	9.75
6.21	3.07	5.93	11.29	6.31	8.19	16.15	8.77	7.79	22.12	11.51	9.48
5.49	2.97	4.60	13.11	7.03	9.26	12.00	5.66	6.48	20.13	10.15	8.74
5.51	2.98	4.52	16.41	8.17	12.21	12.98	6.66	6.52	21.83	10.12	9.35
6.26	3.54	5.44	9.35	4.71	6.71	15.26	7.74	7.80	20.24	11.71	9.05
6.76	4.39	5.21	11.98	5.60	8.84	11.82	5.31	6.23	19.85	8.20	8.94
11.33	11.73	5.30	14.57	9.13	9.15	9.14	4.24	4.82	23.58	13.20	9.93
4.57	2.82	4.18	14.95	11.94	7.68	15.00	7.18	7.72	15.17	6.28	6.43
6.50	6.88	4.41	10.46	7.95	6.08	15.27	7.82	7.79	20.31	9.09	11.90
5.69	3.60	4.40	14.54	7.97	10.91	11.04	5.38	5.78	22.54	13.57	8.91
5.41	3.89	4.33	12.58	8.53	8.15	13.89	6.93	7.06	19.98	7.88	9.12
13.11	14.88	7.49	18.93	18.58	8.53	12.44	5.81	6.31	20.39	9.11	8.39
6.68	5.00	5.52	13.97	8.30	8.63	11.27	5.17	5.55	19.80	8.89	8.19
6.19	5.50	4.26	16.60	13.60	8.45	13.04	6.00	6.80	17.45	8.72	7.65
8.20	7.18	5.07	17.04	14.17	9.48	12.65	5.94	6.11	20.28	9.19	8.32
8.95	*8.95*	*6.15*	*16.00*	*12.90*	*8.89*	11.91	5.58	5.80	19.41	8.51	8.00
6.84	5.83	5.36	12.56	10.48	7.20	12.45	6.20	5.74	29.30	13.02	11.99
p Value [*ppi2 NL vs. rpn8a NL*			0.000	0.003	0.000	p Value [*ppi2 LL vs. rpn8a LL*]			0.000	0.000	0.000
